# SGK1 in Human Cancer: Emerging Roles and Mechanisms

**DOI:** 10.3389/fonc.2020.608722

**Published:** 2021-01-19

**Authors:** Yiwen Sang, Piaoping Kong, Shizhen Zhang, Lingyu Zhang, Ying Cao, Xiuzhi Duan, Tao Sun, Zhihua Tao, Weiwei Liu

**Affiliations:** ^1^ Department of Laboratory Medicine, The Second Affiliated Hospital of Zhejiang University School of Medicine, Hangzhou, China; ^2^ The Cancer Institute of the Second Affiliated Hospital and Institute of Translational Medicine, Zhejiang University School of Medicine, Hangzhou, China

**Keywords:** serum and glucocorticoid-induced protein kinase 1, autophagy, metabolism, tumor microenvironment, therapeutic resistance

## Abstract

Serum and glucocorticoid-induced protein kinase 1 (SGK1) is a member of the “AGC” subfamily of protein kinases, which shares structural and functional similarities with the AKT family of kinases and displays serine/threonine kinase activity. Aberrant expression of SGK1 has profound cellular consequences and is closely correlated with human cancer. SGK1 is considered a canonical factor affecting the expression and signal transduction of multiple genes involved in the genesis and development of many human cancers. Abnormal expression of SGK1 has been found in tissue and may hopefully become a useful indicator of cancer progression. In addition, SGK1 acts as a prognostic factor for cancer patient survival. This review systematically summarizes and discusses the role of SGK1 as a diagnostic and prognostic biomarker of diverse cancer types; focuses on its essential roles and functions in tumorigenesis, cancer cell proliferation, apoptosis, invasion, metastasis, autophagy, metabolism, and therapy resistance and in the tumor microenvironment; and finally summarizes the current understanding of the regulatory mechanisms of SGK1 at the molecular level. Taken together, this evidence highlights the crucial role of SGK1 in tumorigenesis and cancer progression, revealing why it has emerged as a potential target for cancer therapy.

## Introduction

Serum and glucocorticoid-induced protein kinase 1 (SGK1) was initially cloned as an immediate early gene transcriptionally activated by serum and glucocorticoids in rat mammary tumor cells (1, 2). In humans, SGK1 has been found to be expressed in nearly all tissues tested (3); however, its transcript levels vary profoundly among different cell types and tissues (4). In addition, the subcellular localization of SGK1 may depend on the functional state of the cell (5). Ion channels (e.g., ENaC and KCNE1/KCNQ1), carriers (such as NCC and NHE3), Na(+)/K(+)-ATPase, enzymes (including GSK3), and transcription factors or regulators (including forkhead box O 3a (FOXO3a), NF-kappaB, β-catenin and p27) are extensively regulated by SGK1 (6). Thus, SGK1 impacts a wide variety of physiological functions (7) and plays an active role in the pathophysiology of several disorders, including hypertension (8), diabetes (9), inflammation (10), autoimmune disease (11), and tumor growth (12).

SGK1 belongs to the “AGC” subfamily of protein kinases, containing 60 members, including PKA, PKG, and PKC, which all share a conserved catalytic kinase domain (13). And SGK1 shares approximately 54% identity of its catalytic domain with protein kinase B (PKB, also called Akt) (13). Structurally, SGK kinases, as most AGC kinases, consist of three domains: an N-terminal variable region, a catalytic domain, and the C-terminal tail (13). SGK1 is identified and characterized as a tumor-promoting gene, and SGK1 dysregulation has been observed in several types of malignancies, including breast cancer (14), gastric cancer (15), lung cancer (16, 17), and prostate cancer (18). It has essential roles and functions in almost all aspects of tumor occurrence, and progression, including tumorigenesis; cancer cell proliferation, apoptosis, invasion, metastasis, and autophagy; and the response to anti-tumor treatment (4, 6, 12). Due to the multiple functions of SGK1 in human cancer, the downstream target genes and relevant signaling pathways regulated by SGK1 interweave into a huge signaling network.

In recent years, as one of the laboratories studying the role and function of SGK1 in human cancer, we identified SGK1 as a crucial key molecule involved in prostate cancer (PCa) progression through regulation of cell apoptosis, cell cycle, invasion/migration, and autophagy (19, 20). The role of SGK1 in human cancer has been explored in numerous clinical, translational, and basic studies (6, 11, 12). An increasing amount of scientific evidence has confirmed the therapeutic potential of SGK1 in human cancer (11), and a critical review is necessary. In this review, we focus on the diagnostic and prognostic value of SGK1 in human cancer, which has not yet been critically reviewed elsewhere. Furthermore, to further clarify the roles and mechanisms of SGK1 in cancer, we systematically discuss whether SGK1 is an oncogene or a cancer suppressor by backtracking and summarizing the studies covering cells, cancer tissues and biofluids. We further summarize and discuss these findings and the implications for molecular research.

## Clinical Value of SGK1 in Cancer

A growing number of investigations suggest that SGK1 is a potential predictor of tumor type, tumor grade and lymph node metastasis in multiple human cancer types (21). Moreover, SGK1 serves as a prognostic factor for the survival of cancer patients (16, 21, 22).

### SGK1 Expression in Cancer

The expression pattern of SGK1 has been extensively investigated and compared in nontumor and tumor human tissues (**Table 1**). In most tumor types, the expression of SGK1 is dysregulated, although SGK1 upregulation or downregulation has been differentially observed, subject to the particular tumor type. In solid tumors, SGK1 is often upregulated in most of the cancer, such as adrenocortical adenomas (26), breast cancer (14), endometrial cancer (30), gastric cancer (GC) (15), lung cancer (16, 17, 31), medulloblastoma (32), oral squamous cell carcinoma (OSCC) (33), ovarian cancer (34), prostate carcinoma (20, 35), renal clear cell carcinoma (32), and rhabdomyosarcoma (36). Although the exact reason why SGK1 expression in cancer in the intestinal system is downregulated remains unclear (24, 25), it has been postulated to most likely be due to transcriptional repressors acting on the SGK1 promoter (37). What these repressors are and how they are controlled remains unclear (37). Recently, Chen et al. (29) reported that carcinomatous SGK1 expression in human colorectal cancer (CRC) was notably elevated compared with that in nontumor controls according to immunohistochemical assays, which was inconsistent with previous observations (24, 25).

**Table 1:** Expression pattern of SGK1 in different types of human cancer.

Type of Cancer	Functions	References
***Downregulated***		
Adrenocortical carcinoma	Poor overall survival	(23)
Colorectal cancer	Not given	(24, 25)
***Upregulated***		
Adrenocortical adenomas	Tumorigenesis	(26)
Breast cancer	Promoting growth and metastasis	(14)
B-cell lymphoma	Tumorigenesis	(27, 28)
Colorectal cancer	Promoting growth and metastasis	(29)
Endometrial carcinoma	Tumor growth	(30)
Gastric carcinoma	Poor prognosis	(15)
Hodgkin lymphoma	Tumorigenesis	(28)
Lung carcinoma	Promoting growth and metastasis	(16, 17, 31)
Medulloblastoma	Tumorigenesis	(32)
Myeloma	Tumor growth	(28)
Oral squamous cell carcinoma	Promoting growth and invasion	(33)
Ovarian carcinoma	Tumor growth	(34)
Prostate cancer	Promoting growth and metastasis	(20, 35)
Renal clear cell carcinoma	Tumorigenesis	(32)
Rhabdomyosarcoma	Promoting growth and invasion	(36)

Decreased SGK1 expression was also observed in adrenocortical carcinoma (23), which is associated with adrenocorticotropic hormone (ACTH)-independent glucocorticoid secretion (23). In contrast, tumors originating from medullary and lymphatic systems often demonstrate overexpression of SGK1 (27, 38). SGK1 is often upregulated in both acute and chronic myelogenous leukemia, such as in myeloma (39), B-cell lymphoma (27, 28), and Hodgkin lymphoma (38), and has been demonstrated to be significantly correlated with enhancer-associated rearrangements and highly recurrent mutations of the SGK1 gene (27, 28, 38).

### Clinical Significance of SGK1 Tissue Expression in Cancer

As stated above, aberrant expression of SGK1 is closely related to the clinical characteristics of human cancer. Moreover, SGK1 expression was suggested to indicate a later clinical stage, as well as the extent of metastasis. For instance, a high copy number of the SGK1 gene was positively correlated with short-interval recurrence and metastasis in high-grade Müllerian adenosarcoma (40). SGK1 mRNA expression was significantly higher in nonsmall cell lung cancer and was correlated with several clinical features, being elevated in high-grade tumors and in tumors with a larger size and worse clinical stage (31); however, no correlation was found between SGK1 protein expression and these clinical parameters (31). Naruse et al. (33) found that increased SGK1 expression in oral squamous cell carcinoma tissue was significantly associated with tumor stage and pattern of invasion (P<0.05 and P<0.01, respectively). In addition, higher SGK1 expression was observed in 1090 tumor samples of invasive breast cancer from The Cancer Genome Atlas (TCGA) (41). Further study indicated that SGK1 was essential for osteoclastogenesis and dramatically correlated with breast cancer bone metastasis (42). In contrast, Lee et al. (43) demonstrated that SGK1 was upregulated in response to, and an important controller of intestinal cell differentiation. Reexpression of SGK1 in colorectal cancer cell lines resulted in differentiation, decreased migration rates, and inhibition of metastasis (43). In addition, Szmulewitz et al. (18) found that SGK1 expression was high in most untreated prostate cancers and declines with androgen deprivation. However, their data further suggested that relatively low expression of SGK1 is associated with higher tumor grade and increased cancer recurrence (adjusted log-rank test P = 0.077) and is a potential indicator of aberrant AR signaling in these tumors (18). These evidence suggests the significance of SGK1 in terms of its correlation with cancer staging, differentiation, and metastasis. Moreover, these studies indicated that SGK1 expression varies greatly in different type of cancers, and is tumor- and cellular context-dependent.

### The Prognostic Value of SGK1 in Cancer

SGK1 not only has great potential in indicating the clinical features of human cancer but also plays an important role in predicting the progression and prognosis of cancer patients. Abbruzzese et al. (31) showed that SGK1 upregulation in tissue predicted cancer progression and a worse prognosis in nonsmall cell lung cancer (NSCLC) patients. Consistent with this report, Tang et al. (16) demonstrated that high SGK1 expression had strong prognostic value for reduced overall survival (OS) in NSCLC patients. In esophageal squamous cell carcinoma patients, both OS and disease-free survival (DFS) were significantly shorter in the SGK1-high group than in the low group (OS: P = 0.0055; DFS: P = 0.0240) (44). In gastric cancer patients, serum Lnc-SGK1 expression in combination with H. pylori infection was significantly associated with poor prognosis and could be an ideal diagnostic index in human GC (15). Conversely, fewer tumor copy number segments of the SGK1 gene were found to be markedly associated with poor survival in glioblastoma multiforme patients (22). Moreover, increased median overall survival associated with increased SGK1 copy number segments may be a reflection of better tumor oxygenation (22). A similar observation was made in adrenocortical carcinoma patients, Ronchi et al. (23) showed that low SGK1 protein levels were associated with poor overall survival in patients with adrenocortical carcinoma (P < 0.005; hazard ratio = 2.0; 95% confidence interval = 1.24–3.24), independent of tumor stage and glucocorticoid secretion. In prostate cancer patients, Szmulewitz et al. (18) found that high-grade cancers were nearly twice as likely to have relatively low SGK1 staining compared to low-grade cancers (13.8% vs. 26.5%, P = 0.08). In addition, low SGK1 expression in untreated tumors was associated with an increased risk of cancer recurrence (adjusted log-rank test P = 0.077), with 5-year progression-free survival of 47.8% versus 72.6% (P = 0.034) (18). Therefore, SGK1 seems to be an indicator of a good or bad prognosis for cancer patients, depending on the tumor type.

Therapy resistance is a major risk factor for poor prognosis in cancer patients who undergo chemo- and radio-therapy. In prostate cancer, despite new treatments for castration-resistant prostate cancer (CRPC), the prognosis of patients with CRPC remains bleak due to acquired resistance to androgen receptor (AR)-directed therapy. Isikbay et al. (45) found that SGK1 activation plays a crucial role in GR-mediated CRPC progression through acquired resistance to androgen receptor (AR)-directed therapy. In doxorubicin (DOX)-treated rhabdomyosarcoma, SGK1 was found to often be upregulated and led to a poor prognosis (36). In addition, SGK1 was detected to play a key role in the development of resistance to cancer chemotherapy in NSCLC patients (16). Tang et al. (16) demonstrated that high SGK1 expression had strong prognostic value for reduced overall survival in NSCLC patients that received chemotherapy.

## Functional Roles of SGK1 in Cancer

Tumor development is a multistep process that includes sustained proliferation signaling, evasion of growth suppressors, cell death resistance, enabling of replicative immortality, angiogenesis induction, invasion and metastasis activation, energy metabolism reprogramming, evasion of immune destruction, and creation of a “tumor microenvironment” (46). A growing number of investigations indicate that SGK1 has critical roles in tumorigenesis (47), cancer cell proliferation and apoptosis (48, 49), cancer cell invasion (50) and migration (51), cancer cell autophagy (19, 20, 30), cancer metabolism (52, 53), therapeutic resistance (6, 54), and the tumor microenvironment (39, 55). The following section will detail the findings related to the functional roles of SGK1 in diverse human cancers.

### Tumorigenesis

As a critical factor that senses the genomic response to cellular and environmental changes during carcinogenesis, SGKs, including SGK1, are extensively reported to be dysregulated during malignant transformation of normal human tissues (4, 21). Elevated SGK1 is intimately linked to tumorigenesis (21). Several reports have indicated that SGK1 exhibits an oncogenic role in the initiation of human cancer. Wang et al. (56) found that increased SGK1 expression facilitated the development of intestinal tumors in adenomatous polyposis coli (APC)-deficient mice. Feng et al. (57) showed that glucocorticoids elevated during chronic restraint mediate the effect of chronic restraint on p53 through induction of SGK1, which in turn increases MDM2 activity and decreases p53 function, thus providing direct evidence that SGK1 promotes colonic tumorigenesis *in vivo *(57). Using high-resolution single nucleotide polymorphism microarrays (Affymetrix SNP 6.0) to detect copy number alterations (CNAs) related to early adrenocortical adenomas, SGK1 was identified to be involved in tumorigenesis of adrenocortical adenoma (26). Following chemical carcinogenesis, SGK1 knockout mice [sgk1(-/-)] mice developed significantly fewer colonic tumors than wild-type littermates [sgk1(+/+)], suggesting that SGK1 deficiency counteracts the development of colonic tumors, an effect due at least in part to upregulation of FOXO3a and BIM (47). In addition, similar results were obtained in another report, showing that EMD638683, a selective inhibitor of SGK1, significantly decreased the number of colonic tumors following chemical carcinogenesis *in vivo *(58). Therefore, we can conclude that SGK1 governs key processes during the development of various cancer types.

### Cancer Cell Proliferation and Apoptosis

**Figure 1:**
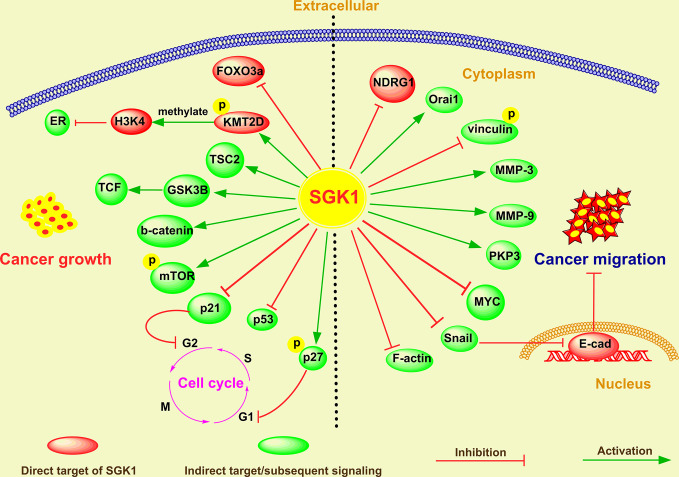
The emerging roles and mechanisms of SGK1 in cancer growth and metastasis.

As a robust cellular regulator of gene expression, SGK1 is involved in a broad range of cell growth signaling pathways through regulation of the expression of downstream genes and or posttranslation modification of proteins, which in turn promotes or suppresses cell proliferation. SGK1 determines cancer cell proliferation or cell apoptosis in various tumors (21, 59, 60). The global regulatory mechanism of SGK1 in determining cancer cell destiny is shown in **Figure 1**.

In breast cancer, Wu et al. (60) reported that glucocorticoid receptor (GR)-mediated induction of SGK-1 expression increased cancer cell proliferation by inactivating FOXO3a and that SGK1 activation remarkably decreased the FOXO3a-induced apoptosis in SK-BR-3 breast cancer cells. Moreover, in hypoxic breast cancer cells, SGK1 expression was obviously stimulated to sustain cell survival (61). SGK1, which is activated by 3-phosphoinositide dependent kinase-1 (PDK1), contributes to the maintenance of residual mTORC1 activity and cancer cell growth through direct phosphorylation and inhibition of tuberous sclerosis 2 (TSC2) (54). Recently, Toska et al. (62) found that PI3K/AKT inhibitors activated ER, which promoted SGK1 transcription through direct binding to its promoter. In addition, elevated SGK1, in turn, phosphorylated lysine (K)-specific methyltransferase 2D (KMT2D), suppressing its function and leading to a loss of methylation of lysine 4 on histone H3 (H3K4) and a repressive chromatin state at estrogen receptor (ER) loci that attenuated ER activity (62). All these evidences suggest that either activation or upregulation of SGK can significantly promote the growth of breast cancer.

SGK1 expression also promotes the development of intestinal tumors in adenomatous polyposis coli (APC)-deficient mice, an effect at least partially due to enhanced beta-catenin protein abundance (56). *In vitro* experiments also revealed that SGK1 overexpression enhanced colonic tumor cell proliferation activity and inhibited cell apoptosis induced by 5-fluorouracil (5-FU), while SGK1 shRNA and inhibitors showed inverse effects (48). Similarly, overexpression of SGK1 markedly promoted the growth of NSCLC cells by promoting the phosphorylation of glycogen synthase kinase-3 beta (GSK3β) and activating beta-catenin/T cell factor (TCF) signaling (17). In glioblastoma, the SGK1 kinase inhibitor SI113 drastically reduced cell viability and clonogenic capabilities *in vitro* and inhibited tumor growth *in vivo *(63, 64). Further research indicated that SI113 treatment caused endoplasmic reticulum stress and apoptosis in endometrial cancer cells, evidenced by cleavage of the apoptotic markers poly-ADP-ribose polymerase (PARP) and Caspase-9 (30). SGK1 inhibitor-induced apoptosis was also reported in lymphocyte predominant (LP) cells and large B-cell lymphoma (DLBCL) cells (38). Recently, we demonstrated that ectopic expression of SGK1 significantly increased cell viability in prostate cancer cells (19). Conversely, SGK1 inhibition mediated by either GSK650394 or SGK1 shRNA induced caspase-dependent apoptosis, evidenced by cleavage of the apoptosis markers caspase-3, 8, and 9 and PARP and by Bax upregulation and Bcl-2 downregulation (19).

SGK1 also plays an important role in cell cycle regulation. SGK1 contributed to cell survival and cell cycle progression *via* downregulation of p53 expression by enhancing its mono- and polyubiquitylation in RKO colorectal cancer cells (65). Hong et al. (66) reported that SGK1 activation mediated p27 T157 phosphorylation and cytoplasmic p27 mislocalization, which in turn promoted G1 phase cell cycle progression in lung cancer. In multiple myeloma (MM), induction of SGK1 expression by the cytokines interleukin (IL)-6, tumor necrosis factor-α (TNF-α), and IL-21 significantly supported the growth of myeloma cells, whereas downregulation of SGK1 with shRNAs resulted in decreased proliferation of myeloma cells and reduced cell numbers (39). On the molecular level, this was reflected by the upregulation of cell cycle inhibitory genes, for example, CDKNA1/p21, whereas positively acting factors, such as CDK6 and RBL2/p130, were downregulated (39). In addition, SI113-mediated cell cycle arrest was widely confirmed in several cancer types, including colon cancer (67), hepatocarcinoma (68) and glioblastoma (69). In line with these reports, we showed that either SGK1 silencing or GSK650394, the first reported specific inhibitor of SGK1 (49), dramatically caused G2/M arrest and activated apoptosis in prostate cancer (19). Mechanistically, SGK1 inhibition upregulated the expression of p21, which is also known as a cyclin-dependent kinase (CDK) inhibitor that can inhibit the formation of the cyclin-CDK complex (19).

Overall, SGK1 can regulate multiple downstream effector molecules related to apoptosis, cell growth and cell cycle through transcriptional regulation and/or phosphorylation, thus participating in the modulation of cancer growth. However, the interaction between these downstream effector molecules needs further study.

### Cancer Cell Invasion and Migration

Aberrant SGK1 expression has been reported in metastatic cancers, which universally display an aggressive pathophysiology (43, 70). Consistent with this, it has been reported that SGK1 is essential for the invasion and metastasis of different human cancers (**Figure 1**). Overexpression of SGK1 significantly promotes cell migration and invasion in various cancers, including breast cancer (42), lung cancer (17), colon cancer (51), glioma (71), hepatoma (71), OSCC (33), prostate cancer (20), and rhabdomyosarcoma (36).

In breast cancer, SGK inhibition significantly impaired cell migration by downregulating N-myc downregulated gene 1 (NDRG1) (50). Bone metastasis is a severe complication associated with various carcinomas. Using an intracardiac injection model in mice, Zhang et al. (42) reported that SGK1 was essential for osteoclastogenesis and promotes breast cancer bone metastasis by regulating the Ca^2+^ release-activated Ca^2+^ channel Orai1; inhibition of SGK1 resulted in a significant reduction in bone metastasis (42).

In colorectal cancer, transfection with a constitutively active SGK1 mutant significantly enhanced cell motility and cell migration *via* vinculin dephosphorylation (51). Liang et al. (48) also found that SGK1 overexpression promoted colonic tumor cell migration, while SGK1 shRNA and inhibitors showed the inverse effects (72). In contrast, Lee et al. (43) showed that SGK1 is upregulated in response to, and an important controller of, intestinal cell differentiation. Overexpression of SGK1 in colorectal cancer cell lines resulted differentiation, decreased migration activity, and inhibition of metastasis in an orthotopic xenograft model (43). These effects may be mediated, at least in part, by SGK1-induced plakophilin 3 (PKP3) expression, an essential component of desmosomes that has been linked to metastatic potential and differentiation in tumours, and increased degradation of MYC (43).

Xiaobo et al. (17) found that overexpression of SGK1 promoted migration of NSCLC cells, while downregulation of SGK1 inhibited migration and metastasis of NSCLC cells. Their further investigation suggested that SGK1 promotes phosphorylation of GSK3 beta, and GSK3 beta phosphorylation induces beta catenin upregulation, which facilitates to upregulate the target genes downstream of beta-catenin/TCF signaling, including genes involved in promoting tumor invasion and metastasis (17).

Recently, we found that SGK1 expression is positively correlated with human prostate cancer progression and metastasis (20). *In vitro*, using wound healing, migration and invasion assays, we showed that SGK1 inhibition significantly attenuates invasion and migration of PCa cells, whereas overexpression of SGK1 dramatically promoted invasion and migration of PCa cells (20). Through an *in vivo* tail vein metastasis assay, we also found that SGK1 downregulation significantly inhibited lung metastasis of prostate cancer cells. Our further results suggested that SGK1 inhibition had antimetastatic effects, evidenced by decreased matrix metalloproteinase 3 (MMP-3) and MMP-9 levels, which were induced at least partially by repression of epithelial-to-mesenchymal transition (EMT) through downregulation of Snail (20).

Several inhibitors of SGK1 have shown great potential in inhibiting tumor metastasis. Using glioblastoma multiforme, hepatocarcinoma and colorectal carcinoma cell lines, Abbruzzese et al. (71) recognized an inhibitory effect of SI113 on cell migration, invasion, and epithelial-to-mesenchymal transition. In addition, when exposed to SI113, these cancer cells showed a remarkable subversion of the cytoskeletal architecture, characterized by F-actin destabilization, phospho-FAK delocalization, and tubulin depolymerization (71). In addition, administration of EMD638683 - an inhibitor specific for SGK1 - decreased the viability of RD and RH30 cells and enhanced the effects of the cytotoxic drug doxorubicin (36). We obtained similar results in PCa cells treated with GSK650394 (20), the first developed inhibitor of SGK (49). Recently, a new SGK1 inhibitor analog was developed, the GSK650394 analog QGY-5-114-A, which significantly inhibited CRC cell migration *in vitro *(72).

These studies indicate that SGK1 can regulate multiple downstream effector molecules involved in EMT and cell migration, thus participating in tumor invasion and metastasis. However, the direct downstream target genes of SGK1 regulating invasion and metastasis need to be further identified.

### SGK1-Mediated Autophagy in Cancer Progression

**Figure 2:**
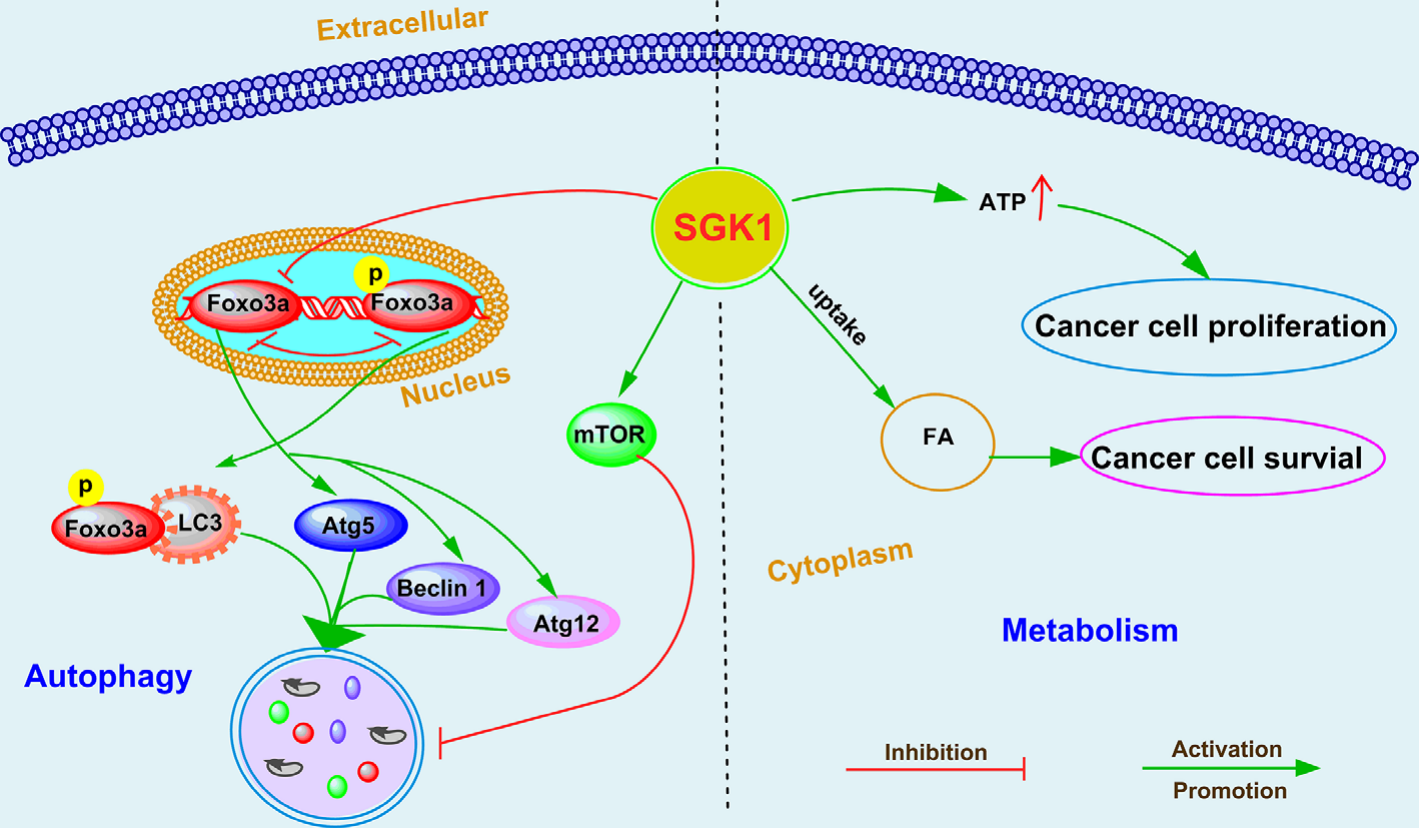
The emerging roles and mechanisms of SGK1 in cancer cell autophagy and metabolism.

Macroautophagy regulation is now recognized as one of the hallmarks of cancer cells (73). Accumulating evidence suggests that autophagy plays a critical role in the various stages of tumorigenesis and tumor progression (74). Depending on the type of cancer and the context, macroautophagy can be a tumor suppressor or it can help cancer cells overcome metabolic stress and the cytotoxicity of chemotherapy (75). SGK1-mediated autophagy modulation was first found in maintenance of skeletal muscle homeostasis (76). Andres-Mateos et al. (76) reported that SGK1 regulated muscle mass maintenance *via* downregulation of proteolysis and autophagy and by increasing protein synthesis during hibernation. In recent years, an increasing number of studies have suggested that SGK1-mediated autophagy regulation plays an important role in the occurrence and progression of cancer (**Figure 2**), including glioblastoma multiforme (63, 69), endometrial cancer (30), and prostate cancer (19, 20). SI113-mediated SGK1 inhibition markedly induced cytotoxic autophagy in human glioblastoma multiforme cells (69). A similar effect was observed in endometrial cancer after treatment with SI113, revealed by an increase in the markers LC3B-II and beclin I, detected *via* both immunofluorescence and western blotting analysis (30). Our recent studies further demonstrated that SGK1-mediated autophagy cross talks with cell death (19) and cell migration (20) in PCa. We demonstrated that SGK1 inhibition, mediated by either GSK650394 or SGK1 shRNA, significantly induced cytotoxic autophagy (19). However, 3MA-mediated autophagy inhibition attenuated SGK1 inhibition-induced apoptosis and antimetastatic effects (19, 20). Moreover, suppression of mTOR and FOXO3a phosphorylation is critical for blockade of the SGK1-induced cytocidal and antimetastatic effects, at least partially *via* pFOXO3a (S253)-LC3 and pFOXO3a(S253)-p27 interactions (19, 20). Although the role and function of SGK1 in regulating autophagy have been confirmed, the key downstream molecules that regulate autophagy need to be further clarified.

### Cancer Metabolism

Several studies have suggested that SGK1 plays a crucial role in the metabolic switch of diverse cell types, including regulating fat and glucose metabolism in adipocytes (77), affecting glucose absorption in the intestine (78), and modulating glycogen metabolism in the muscle/liver/brain (79). For cancer cells to survive during metastasis, they must overcome anoikis, a caspase-dependent cell death process triggered by extracellular matrix (ECM) detachment, and rectify detachment-induced metabolic defects that compromise cell survival (80, 81). Mason et al. (53) identified that SGK1 activation was sufficient to promote ATP production and cell survival during ECM detachment in breast cancer and colorectal cancer. Interestingly, constitutively active SGK1 did not influence caspase activation in either ECM-attached or -detached cancer cells, suggesting that the effects of SGK1 activation on ATP generation and viability are independent of anoikis (53). Unsaturated fatty acids (FAs) are indispensable for cancer cell growth, but to date, the mechanism of increased FA uptake in hypoxia is largely unknown. Matschke et al. (52) showed that exposure to acute or chronic cycling hypoxia markedly upregulated the expression of SGK1, increased uptake of FAs, and increased sensitivity to serum deprivation in NCI-H460 NSCLC cells. In addition, SKG1 inhibition dramatically decreased long-term survival and potently sensitized the parental and anoxia-tolerant NCI-H460 cells to the cytotoxic effects of ionizing radiation under normoxia and the anoxia-tolerant cancer cells under severe hypoxia (52). Although the key roles of SGK1 in cancer metabolism have been identified (**Figure 2**), the underlying mechanisms of SGK1 involvement in metabolism regulation need to be further investigated in cancer.

### Chemo- and Radio-Resistance

**Figure 3:**
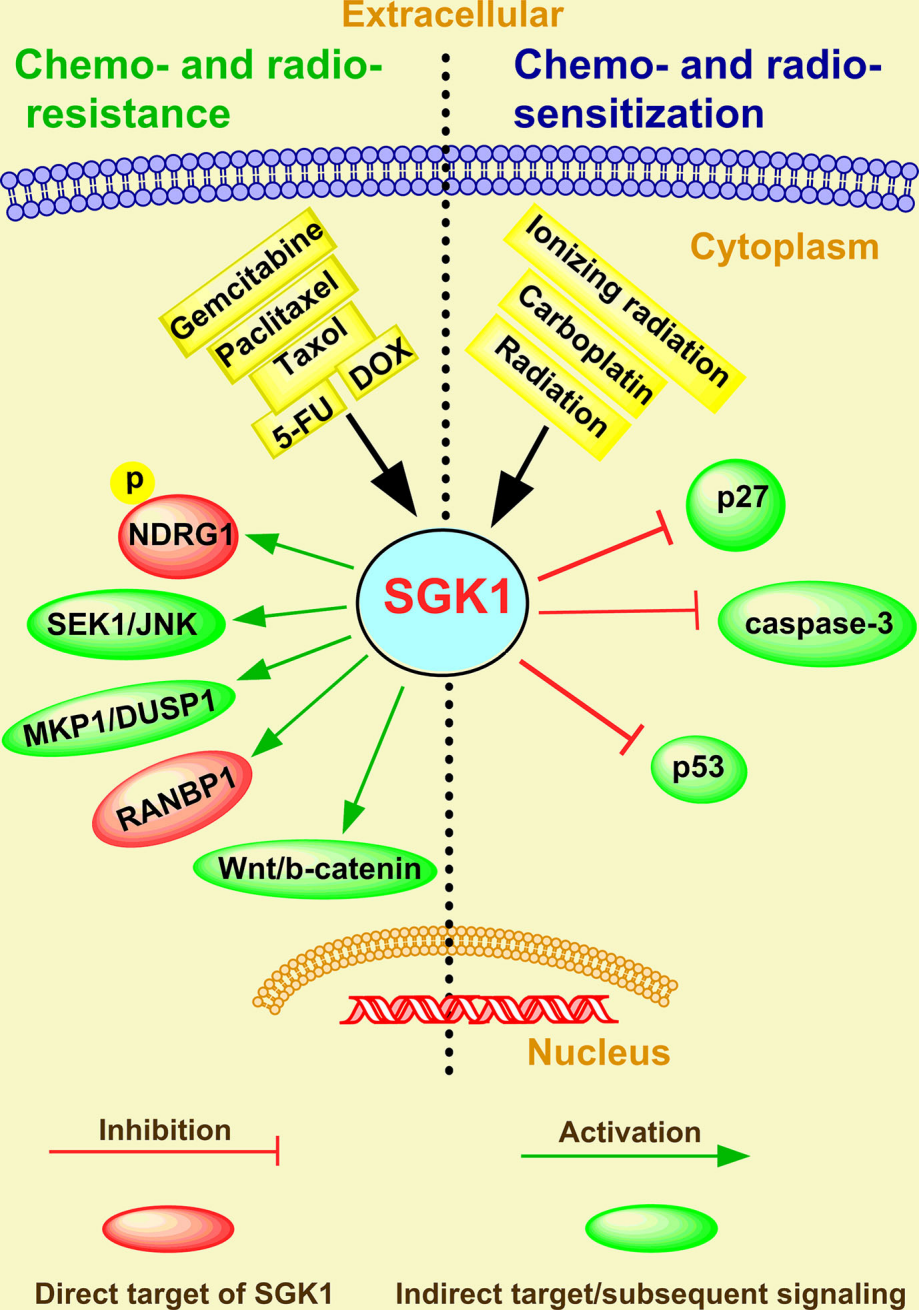
The emerging roles and mechanisms of SGK1 in cancer chemo- and radio-resistance.

Therapeutic resistance of cancer is an important factor in improving the invasiveness and metastasis of cancer cells (82, 83), which depends upon escaping apoptosis and increasing drug efflux (84). In the last two decades, several studies have focused on AKT as an important mediator of cancer therapeutic resistance (85). Indeed, a great number of clinical trials evaluating the therapeutic efficacy of AKT inhibitors for cancer therapy have been designed or are ongoing globally (86). However, resistance to these inhibitors has been observed, typically in tumors characterized by SGK1 activation (54, 62, 87, 88). Therefore, SGK1 appears to be a malevolent player in the stress response to chemical and radio-therapeutics and might be responsible for a selective advantage that favors uncontrolled cancer progression and selection of the most aggressive clones (89). Although the role of SGK1 in cancer therapy resistance was reviewed and discussed in 2016 (6), emerging functions and mechanisms have been reported in recent four years. In addition, SGK1 is commonly perceived to be a new drug target candidate for cancer treatment (**Figure 3**).

#### Chemo-Resistance

In breast cancer, Sommer et al. (90) revealed a number of Akt-inhibitor-resistant lines displaying markedly elevated SGK1 and that exhibited significant phosphorylation of the SGK1 substrate NDRG1. In contrast, most Akt-inhibitor-sensitive cell lines display low/undetectable levels of SGK1 (90). In tamoxifen-resistant human breast cancer, immunohistochemical staining analysis showed an inverse correlation between SGK1 expression and the severity of tamoxifen resistance (91). Moreover, SGK1 depletion prevented the dexamethasone-induced increase in SGK1 expression and the inhibitory effects of dexamethasone on paclitaxel-induced SEK1-JNK signaling and apoptosis in MDA-MB-231 breast cancer cells (92).

In ovarian carcinoma, *in vitro* assays showed that in glucocorticoid receptor (GR)-positive HeyA8 and SKOV3 cells, dexamethasone (100 nM) treatment upregulated the pro-survival genes SGK1, and MKP1/DUSP1 and inhibited carboplatin/gemcitabine-induced cell death (93). However, in a preclinical model of ovarian cancer, the SGK1 inhibitor SI113 counteracted the development of paclitaxel resistance and restored drug sensitivity (94).

In colorectal cancer, SGK1 overexpression promoted colonic tumor cell proliferation and migration and inhibited cell apoptosis induced by 5-fluorouracil (5-FU) (48). SGK1 also enhanced the transcript levels RAN-binding protein 1 (RANBP1), a major effector of the GTPase RAN, and decreased Taxol sensitivity in RKO colon carcinoma cells (95). However, SI113-mediated SGK1 inhibition appears to be effective in inducing cell death in RKO cells and potentiating paclitaxel sensitivity, indicating that this new molecule could be efficiently employed, alone or in combination with paclitaxel, in colon cancer chemotherapy (67). Moreover, knockdown of SGK1 significantly decreased doxorubicin resistance in colorectal cancer (96). In addition, through comprehensive analysis of a microRNA-messenger RNA regulatory network, Zhang et al. (97) found that SGK1 drove gemcitabine-resistance in gemcitabine-resistant bladder cancer cells. All these studies indicate that SGK1 plays a key role in tumor chemotherapy resistance and is a promising therapeutic target of cancer.

#### Radio-Resistance

In addition to chemo-resistance, increasing studies have shown that SGK1 can also facilitate chemoresistance. Thus, combining SGK1 inhibition with radiation therapy may provide a powerful novel anti-tumor strategy to combat cancer. Towhid et al. (58) reported that pharmacological inhibition of SGK1 with EMD638683 (50 µM) synergized with low doses of radiation (3 GY) caused mitochondrial depolarization and late apoptosis (necro-apoptosis) in a colon carcinoma (CaCo-2) cell line, with a relative increase in caspase-3, indicating that EMD638683 promotes radiation-induced suicidal death of colon tumor cells. SKG1 inhibition also decreases long-term survival and potently sensitized the parental and anoxia-tolerant NCI-H460 cells under normoxia and anoxia-tolerant lung cancer cells under severe hypoxia to the cytotoxic effects of ionizing radiation (52). The role of SGK1 in the development of radio-resistance of liver cancer and glioblastoma multiforme was extensively investigated by Talarico et al. (63, 68, 69). Consistent with knockdown and overexpression cellular models for SGK1, SI113 potentiated and synergized with radiotherapy in killing liver tumor cells without toxicity, which was confirmed by a short-term *in vivo* toxicity test (68). They further showed that SGK1 was overexpressed in highly malignant gliomas and that SI113 dramatically potentiated the effects of radiotherapy, modulated the response to oxidative stress, and induced cytotoxic autophagy in glioblastoma multiforme cells (63, 69). Recently, Chen et al. (98) observed a CD44+ cancer stem cell (CSC) population increase in radioresistant LNCaP (LNCaPR18) and C4-2 (C4-2R26) PCa cells compared with respective parental cells. In addition, they suggested that higher GR levels and SGK1-Wnt/β-catenin signaling activation contributed to the radiation-induced CSC increase in PCa (98). All these investigations indicate that SGK1 could be a promising therapeutic target for overcoming radio-resistance in human cancer.

### The Regulatory Effect of SGK1 on the Tumor Microenvironment

**Figure 4:**
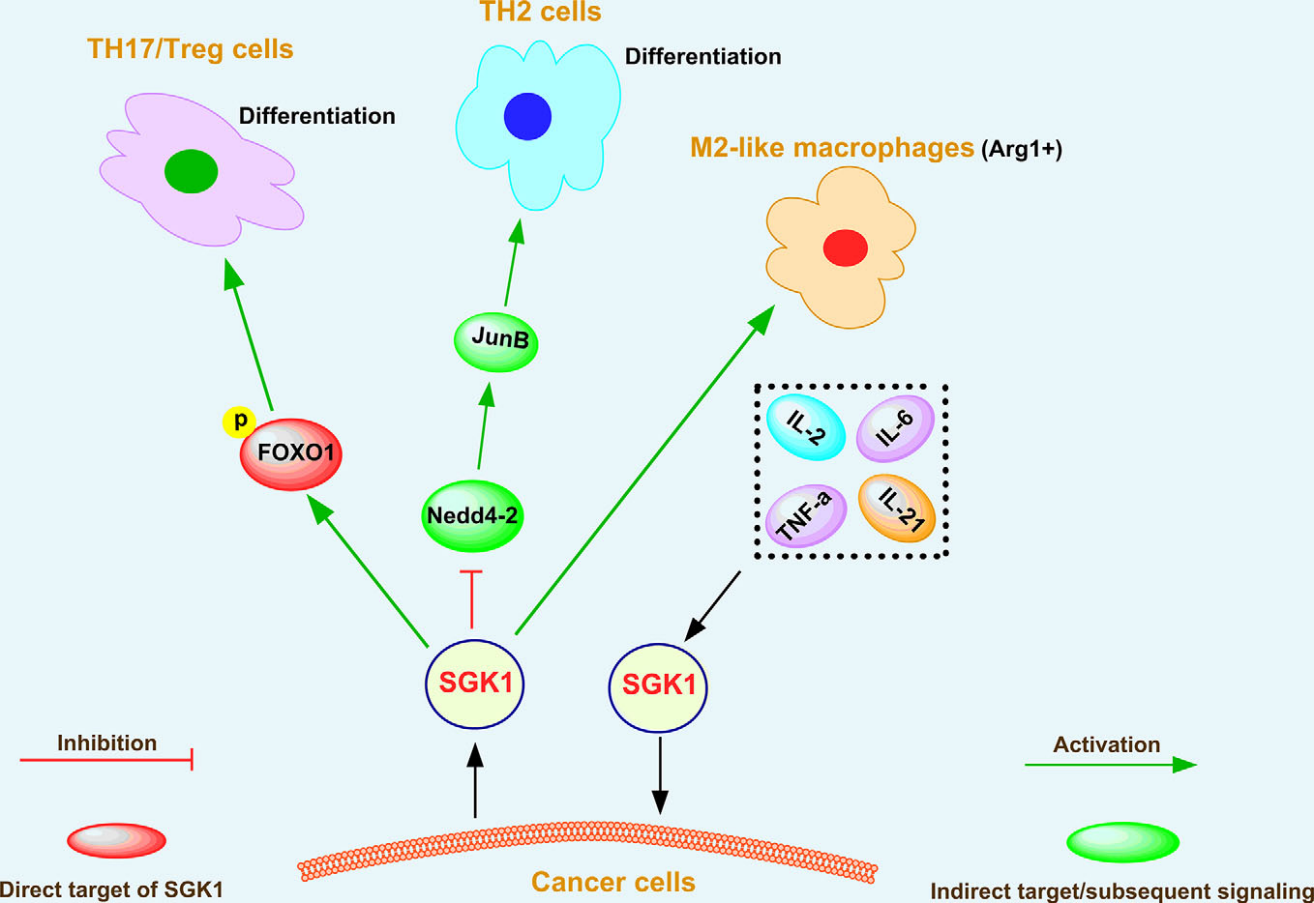
The regulatory effect of SGK1 on the tumor microenvironment.

The tumor microenvironment plays a crucial role in cancer initiation (99), progression (100), and metastasis (101). The primary components of the tumor microenvironment are fibroblasts (102), immune cells (103), endothelial cells (104), ECM (105), and cytokines (106). Among them, immune cells play vital roles in enhancing cancer progression by secreting numerous pro-inflammatory factors (107). According to several reports (15, 55, 108, 109), SGK1 can modulate the function of immune cells, such as T helper (TH) cells, regulatory T cells (Tregs), and tumor-associated macrophages (TAMs), and plays an important role in the tumor microenvironment through crosstalk with cytokine signaling pathways (39, 110) and chemokine signaling pathways (111), as shown in **Figure 4**.

Heikamp et al. (55) found that T cells with selective knockout of SGK1 were resistant to experimentally induced asthma, generated substantial IFN-γ in response to viral infection and more readily rejected tumors. Further investigations indicated that SGK1 could promote T helper type 2 (TH2) differentiation by negatively regulating degradation of the transcription factor JunB mediated by the E3 ligase Nedd4-2 (55). Simultaneously, SGK1 restrained the production of interferon-γ (IFN-γ) by increasing the expression of the long isoform of the transcription factor TCF-1, thus promoting cancer growth (55). Therefore, SGK1 downregulation contributes to tumor rejection *via* blocking TCF-1 signaling. SGK1 and the phosphorylation and translocation of the downstream transcription factor forkhead box O 1 (FOXO1) also play critical roles in differentiation of T helper 17 cells/regulatory T cells (TH17/Treg) (108). In addition, tumor-associated macrophages (TAMs) have attracted attention because they can regulate key cancer-related activities (112). In growing tumors, TAMs are often referred to as M2-like macrophages (113), which are cells that display tumorigenic and immunosuppressive functions and express the enzyme arginase 1 (Arg1) (114). Recently, Arlauckas et al. (109) showed that Arg1+ macrophages are more abundant in tumors than in other organs, and SGK1 was recognized as a novel marker of Arg1+ macrophages, indicating its critical role in the function of Arg1+ macrophages.

SGK1 was also demonstrated to be a downstream effector of interleukin-2 (IL-2) in kidney cancer (110). IL-2 is a cytokine that is essential for lymphocytic survival and function (115). Amato et al. (110) showed that IL-2 binding to its receptor triggers survival signal transduction pathways contributing to A-498 kidney cancer cell proliferation *via* SGK1 activation. Fagerli et al. (39) determined the changes in gene expression induced by IL-6, TNF-α, IL-21, or coculture with bone marrow stromal cells in myeloma cell lines. Among a limited set of genes that were consistently activated in response to growth factors, SGK1 was identified as a prominent transcriptional target of cytokine-induced signaling in myeloma cells, indicating that SGK1 is a highly cytokine-responsive gene and promotes malignant growth of myeloma cells (39). On the one hand, these studies indicate that SGK1 plays an important role in the maturation of immune cells and their antitumor effects; on the other hand, SGK1 functions as an oncogene in promoting tumor growth *via* crosstalk with cytokines.

## Regulation of SGK1 in Human Cancer

**Figure 5:**
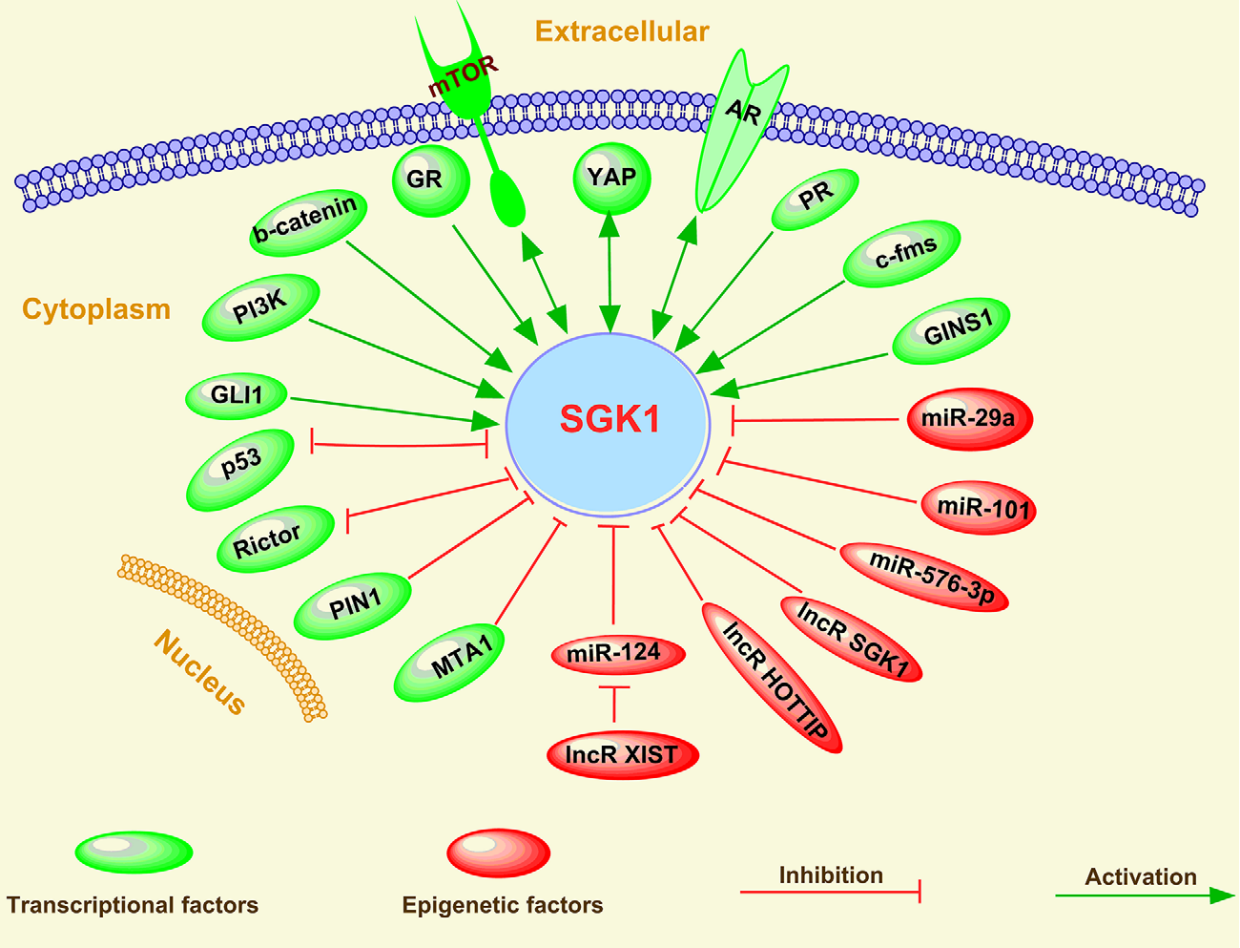
Regulation of SGK1 in human cancer.

Although SGK1 plays multiple roles in cancer initiation, progression, metastasis, and treatment response in humans, its expression is modulated by various factors, including a multitude of stimuli, such as growth factors, mineralocorticoids, and cytokines. Moreover, various cellular stresses, such as hyperosmotic cell shrinkage, heat shock, ultraviolet irradiation, and oxidative stress, have been shown to induce SGK1 gene transcription (4, 12). Mechanistically, the expression of SGK1 can be regulated *via* both transcriptional factors and epigenetic factor-induced mechanisms in cancer cells (**Figure 5**).

### Transcriptional Factors

Various transcriptional factors, including activators and repressors, have been found to be involved in the regulation of SGK1 expression in human cancer, which has been reviewed above in this study. SGK1 expression regulation by several transcriptional activators, such as mTOR (19, 66, 116–118), AR (45, 51, 119–121), PI3K (53, 122, 123), GR (45, 60, 98, 120, 124), Wnt/β-catenin (45, 125, 126), c-fms (127), Yes-associated transcriptional regulator (YAP) (128), Src (129), progesterone receptor (PR) (130), GINS complex subunit 1(GINS1) (131), and GLI family zinc finger 1 (GLI1) (132), and by transcriptional repressors, such as p53 (57, 65, 133), peptidylprolyl cis/trans isomerase, NIMA-interacting 1 (PIN1) (91), Rictor (134, 135), and metastasis associated 1 (MTA1) (61), suggests some of the underlying mechanisms of the oncogenic or tumor suppressive role of these transcriptional factors in human cancer. Therefore, due to the multiple upstream transcriptional activators of SGK1, single inhibition of the activity of one activator usually induces upregulation or functional activation of other activators (45, 54, 136), which is also the main reason for single target drug resistance. For example, several reports have indicated that GR activity was enhanced after therapeutic inhibition of AR signaling (136), which contributed to tumor-promoting PCa cell viability (45). A similar tumor growth bypass pathway was also found in breast cancer; Castel et al. (54) showed that PDK1-SGK1 signaling sustained AKT-independent mTORC1 activation and confers resistance to PI3Kα inhibition, indicating that either PDK1 or SGK1 blockade prevents mTORC1 activation and restores the antitumoral effects of PI3Kα inhibition in resistant cells.

Furthermore, several feedback pathways exist between SGK1 and some of the transcriptional factors mentioned above, including negative feedback pathways between SGK1 and p53 (16, 65), PI3K (62), and Rictor (134, 135) and positive feedback pathways between SGK1 and YAP (128) and AR (121). In addition, Marzook et al. (61) discovered that MTA1 is a novel corepressor of SGK1. Surprisingly, this regulatory corepressive function of MTA1 was lost under hypoxia, allowing enhanced SGK1 expression and engaging the MTA1-SGK1 axis for the benefit of cancer cell survival (61).

### Epigenetic Factors

Epigenetic factors have also been reported to mediate the transcriptional machinery of SGK1. Among various epigenetic factors, miRNAs and lncRNAs have been reported to modulate SGK1 expression.

Using microarray and RNA-Seq-based gene expression profiling and ChIP-Seq analyses of breast cancer cells, Godbole et al. (130) observed that SGK1 and the tumor metastasis-suppressor gene NDRG1 are upregulated and that the microRNAs miR-29a and miR-101-1 targeting the 3’-UTR of SGK1 are downregulated in response to progesterone. SGK1 was also reported as a direct target of miR-576-3p, and miR-576-3p significantly inhibited lung adenocarcinoma migration and invasion by binding to the 3’ untranslated region (3’-UTR) of SGK1 (137).

In nonsmall-cell lung cancer, C-containing genotypes of MIAT rs1061451 were found to be protective factors in NSCLC, and myocardial infarction associated transcript (MIAT), which may act as a ceRNA *via* miR-133a-5p, modulated the SGK1 expression level (138). A similar observation was reported in CRC; lncRNA X-inactive specific transcript (XIST) was shown to positively regulate SGK1 expression by interacting with miR-124 in doxorubicin-resistant CRC cells (96). Moreover, an SGK1-depletion-elicited decrease in DOX resistance was greatly restored by XIST overexpression or miR-124 inhibition in DOX-resistant CRC cells (96).

In human gastric cancer, serum Lnc-SGK1 expression in T cells in combination with H. pylori (Hp) infection and/or a high-salt diet (HSD) was associated with poor prognosis of GC patients (15). Further results indicated that Hp infection and a high-salt diet can upregulate SGK1 expression and in turn enhance Lnc-SGK1 expression through JunB activation (15). In addition, expression of Lnc-SGK1 can induce TH2 and TH17 cells and reduce TH1 cell differentiation by enhancing SGK1 transcription through a cis regulatory mode (15). In contrast, using a luciferase reporter assay, Liu et al. (139) found that the long noncoding RNA HOTTIP decreased expression of the SGK1 gene. Subsequently, they verified that HOTTIP may be an oncogene and that knockdown of HOTTIP inhibited CRC cell proliferation and migration and induced apoptosis by targeting SGK1 (139). All these investigations indicate that miRNAs are the main epigenetic regulators to repress SGK1 expression *via* targeting its 3’-UTR, thus affecting the occurrence and progress of cancer.

## SGK1 Inhibitors

In light of the emerging evidence highlighting multiple roles for SGK1 in mediating tumorigenesis and progression, several specific and selective inhibitors of SGK1 have been developed, including GSK650394 (49), EMD638683 (140), SI113 (141), QGY-5-114-A (72), and ZINC00319000 (142). The mentioned SGK1 inhibitors and their various anti-tumor effects are summarized in **Table 2**.

**Table 2:** SGK1 inhibitors and their various anti-tumor effects.

Molecule	Anti-tumor effects	References
GSK650394	Inducing apoptosis and cell cycle arrest; impairing invasion and migration	[19, 20, 49]
EMD638683	Inducing apoptosis and impairing migration	[30, 140, 143]
SI113	Inducing ER stress and apoptosis; inhibiting EMT	[24, 67, 141]
QGY-5-114-A	Inducing apoptosis and cell cycle arrest; impairing migration	[72]
ZINC00319000	Unclear, it needs to be further investigated	[142]

EMT, epithelial-to-mesenchymal transition; ER stress, endoplasmic reticulum stress.

### GSK650394

Given that SGK1 expression is required for androgen-dependent growth of prostate cancer cells, Sherk et al. (49) developed a novel heterocyclic indazole derivate, GSK650394, which is the first reported SGK1 inhibitor that restrains the enzymatic activity of SGK1, with half maximal inhibitory concentration (IC_50_) values of 62 nM based on an *in vitro* scintillation proximity assay (SPA). GSK650394 functionally inhibited SGK1 in an LNCaP growth assay, with an IC_50_ value of approximately 1 μM (which is similar to the previously measured IC_50_ in other cell-based assays) (49). More recently, GSK650394 has shown anti-tumor effects in several other tumors, including breast cancer (143), squamous cell carcinoma of the head and neck (143, 144), lung cancer (52), CRC (72), and cervical cancer (145). In addition, our results also indicated that GSK650394 could significantly inhibit PCa cells proliferation, invasion and migration (19, 20). However, GSK650394 also inhibits the enzymatic activity of SGK2 in the SPA assay, with an IC_50_ value of 103 nM, indicating that GSK650394 is equally active toward both SGK1 and SGK2. In addition, this inhibitor is only approximately 30-fold more selective for SGK1 than for Akt and other related kinases, such as protein kinase B isoforms (AKT1, AKT2, AKT3), Janus kinase isoforms (JAK1, JAK3), insulin-like growth factor1 receptor (IGF1R), Rho-associated protein kinase (ROCK), dual-specificity tyrosine phosphorylation-regulated kinase (DYRK1A) and PDK1, and less than 10-fold more selective for SGK1 than for Aurora and c-Jun N-terminal kinase (49, 146).

### EMD638683

The second developed SGK1 inhibitor is a benzohydrazide derivative named EMD638683 developed by Ackermann et al. (140). *In vitro* testing identified EMD638683 as an SGK1 inhibitor with an IC_50_ of 3 μM in human cervical carcinoma HeLa cells. Originally, MD638683 served as a template for drugs counteracting hypertension in individuals with type II diabetes and metabolic syndrome *in vivo *(140) but was more recently evaluated in experimental models of colon cancer (58), breast cancer (143), and rhabdomyosarcoma (36). The molecule has been shown to induce pro-apoptotic responses (143); enhance the effects of the cytotoxic drug doxorubicin, leading to reduced migration and decreased cell proliferation (36); and promote radiation-induced suicidal death of colon tumor cells *in vitro* and decrease the number of colonic tumors following chemical carcinogenesis *in vivo *(58). Ackermann et al. (140) demonstrated that EMD638683 also has an inhibitory effect on mitogen- and stress-activated protein kinase 1 (MSK1), cAMP-dependent protein kinase (PKA), protein kinase C-related kinase 2 (PRK2), and the SGK isoforms SGK2 and SGK3. The specificity at a dose of 1 µM has been well tested among these kinases (140). However, in oncological reports, the molecule was tested at a dose of 50 µM (58), and therefore, it is not impossible that other kinases are inhibited at this dose range.

### SI113

What’s more, Ortuso et al. (141) screened a family of dual SRC/ABL small molecule inhibitors characterized by a substituted pyrazolo [3,4-*d*] pyrimidine scaffold to inhibit SGK1 and AKT kinase activity. Among these molecules, SI113 was particularly selective in inhibiting SGK1 kinase activity, while being much less effective in inhibiting AKT1 (141). In addition, a dose-dependence curve of SI113-dependent SGK1 and AKT1 inhibition showed that the inhibition of SGK1 activity occurred at an IC_50_ value of 600 nmol/L, with a 100-fold selectivity compared to AKT1 (against which SI113 had an IC_50_ value equal to 50 µmol/L) (141). Subsequent studies have shown that SI113 induced cell death, thus counteracting cell proliferation in various cancer cell lines (30, 67). Specifically, SI113 induced cell apoptosis, both alone and synergistically with paclitaxel in RKO cells (67) and ovarian cancer cells (94), or synergized with radiotherapy in hepatocarcinoma models *in vitro* and *in vivo *(68). Remarkably, multiple studies have confirmed that SI113 exhibits powerful anti-tumor effects in glioblastoma multiforme, including activating cell apoptosis, induction of endoplasmic reticulum stress, inhibition of epithelial-to-mesenchymal transition, and especially stimulation of cytotoxic autophagy (63, 64, 69, 71, 147). All these studies indicate that SI113 has strong anti-tumor activity, and its selective inhibitory effect on SGK1 is significantly superior to that of the two previously reported inhibitors.

### QGY-5-114-A

More recently, Liang et al. (72) designed and synthesized 39 new analogs of the SGK1 inhibitor GSK650394. They showed that certain analogs, numbered MH-1-11-A, MH1-11-B, QGY-5-90, QGY-OMe, QGY-5-163, QGY-5-114-A, and QGY-5-121, could inhibit the viability of colonic tumor HCT116 cells. To compare the inhibitory potency of these seven analogs, IC_50_ values were determined, and only compound QGY-5-114-A showed a significantly lower IC_50_ value of 122.9 μM, indicating better inhibitory potency than the inhibitor GSK650394. Furthermore, QGY-5-114-A could dramatically restrain colonic tumor cell proliferation *via* activating cell apoptosis and inducing cell cycle arrest at G0/G1 phase, and significantly impede colonic tumor cell migration *in vitro *(72).

### ZINC00319000

Using a structure-based virtual high-throughput screening strategy, Mohammad et al. (142) found four compounds bearing appreciable binding affinity and specificity towards the binding pocket of SGK1. Among them, the compound ZINC00319000 was selected and identified based on docking results with all-atom molecular dynamics simulation for 100 ns. Molecular dynamics simulation results further suggested that the binding of ZINC00319000 stabilizes the SGK1 structure and leads to few conformational changes, indicating that ZINC00319000 might be further exploited as a scaffold to develop promising inhibitors of SGK1 for therapeutic management of associated diseases, including cancer (142). However, its anti-tumor effects and mechanisms need to be further investigated.

## Conclusions

In studies of several tumor types, the expression and function of SGK1 are quite different. This may be due to the specific immune and metabolic microenvironment, and proteogenomics profile of specific tumor type. Although the biological functions and mechanisms of SGK1 have been extensively studied, further studies on its clinical application are still needed. Although several SGK1 inhibitors have paved the way for novel therapeutic interventions in the future, the prospects for clinical application of these inhibitors in cancer therapy are vague, which means that more preclinical studies, especially on toxicity and safety, should be conducted.

SGK1 is involved in the development of almost all tumors and may function as a promising biomarker for cancer diagnosis and prognosis. This review highlights the scientific achievements of human cancer research on SGK1, outlines the advances, and challenges in application of SGK1 as a diagnostic and prognostic tool in cancer, and discusses its biological function and clinical insights. SGK1 functions as an oncogene in some tumors and as a tumor suppressor in others, which suggests that the function of SGK1 is tumor- and cellular context-dependent. Its biological functions encompass tumor occurrence, progression, and metastasis; cell autophagy, metabolism, and therapy resistance; and the tumor microenvironment, indicating its potential as a new target for cancer treatment. Despite the role of SGK1 in cancer has been widely studied, its role and molecular regulation mechanism in tumor autophagy and metabolism need to be further investigated, especially the key downstream effector molecules and possible signaling pathways of SGK1 modulating autophagy and metabolism.

## Author Contributions

YS and PK finished the manuscript and abstract SZ and LZ consulted relevant literatures and completed English revision YC XD and TS completed the figures and tables WL and ZT provided constructive feedback and guidance WL completed critical revisions and proofread the manuscript All authors contributed to the article and approved the submitted version.

## Funding

This study was supported by grant from the National Natural Science Foundation of China Youth Science Foundation Project (Grant nos 81802571 and Zhejiang Medical and Health Science and Technology Project (2019RC039).

## Conflict of Interest

The authors declare that the research was conducted in the absence of any commercial or financial relationships that could be construed as a potential conflict of interest.

## Abbreviations

ACTH, adrenocorticotropic hormone; Arg1, arginase 1; Akt, protein kinase B; APC, adenomatous polyposis coli; AR, androgen receptor; ATP, adenosine triphosphate; BPH, benign prostatic hyperplasia; CDKN1B/p27, cyclin-dependent kinase inhibitor 1B; CNAs, copy number alterations; CRC, colorectal cancer; CSC, cancer stem cell; DC, dendritic cell; DFS, disease-free survival; DOX, doxorubicin; EOC, epithelial ovarian cancer; ER, estrogen receptor; ERα, estrogen receptor α; EC, endometrial cancer; ECM, extracellular matrix; FA, fatty acid; FoxO, forkhead box O; FOXP2, forehead box protein P2; FVPTC, follicular variant of papillary thyroid carcinoma; GINS1, GINS complex subunit 1; GLI1, GLI family zinc finger 1; GR, glucocorticoid receptor; GSK3β, glycogen synthase kinase-3 beta; GNAI2, G protein alpha inhibiting activity polytide 2; H3K4, lysine 4 on histone H3; HCC, hepatocellular carcinoma; HIPK2, homeodomain-interacting protein kinases 2; H. pylori, Helicobacter pylori; IL-2, interleukin-2; JAK, Janus kinase; KMT2D, lysine (K)-specific methyltransferase 2D; LP, lymphocyte predominant; miRNA, smicroRNAs; MM, multiple myeloma; MMPs, matrix metalloproteinase; MTA1, metastasis-associated 1; mTOR, mammalian target of rapamycin; NDRG1, N-myc down-regulated gene 1; NSCLC, non-small cell lung cancer; OS, overall survival; OSCC, oral squamous cell carcinoma; PARP, poly (ADP-ribose) polymerase; PCa, prostate cancer; PDK1, 3-phosphoinositide dependent kinase-1; PIN1, peptidylprolyl cis/trans isomerase, NIMA-interacting 1; PI3K, phosphoinositide 3-kinase; PKP3, plakophilin 3; PR, progesterone receptor; PRAD1, parathyroidadenomatosisgene-1; PTC, papillary thyroid carcinoma; PTEN, phosphatase and tensin homology deleted on chromosome ten; TAM, tumor-associated macrophage; TCF, T cell factor; TCGA, The Cancer Genome Atlas; TH, T helper; TIMP3, tissue inhibitor of metalloproteinases 3; TNF-α, tumor necrosis factor-α; TRAIL, tumor necrosis factor-related apoptosis-inducing ligand; Treg, regulatory T cells; TSC2, tuberous sclerosis 2; XIST, X-inactive specific transcript; YAP, Yes-associated protein; 3’-UTR, 3’-untranslated region; 5-FU, 5-fluorouracil.

## References

[1] Webster MK Goya L Firestone GL Immediate-early transcriptional regulation and rapid mRNA turnover of a putative serine/threonine protein kinase. J Biol Chem (1993) 268:11482–5. 8505283

[2] Webster MK Goya L Ge Y Maiyar AC Firestone GL Characterization of sgk, a novel member of the serine/threonine protein kinase gene family which is transcriptionally induced by glucocorticoids and serum. Mol Cell Biol (1993) 13:2031–40. 10.1128/MCB.13.4.2031 PMC359524 8455596

[3] Waldegger S Barth P Raber G Lang F Cloning and characterization of a putative human serine/threonine protein kinase transcriptionally modified during anisotonic and isotonic alterations of cell volume. Proc Natl Acad Sci USA (1997) 94:4440–5. 10.1073/pnas.94.9.4440 PMC20741 9114008

[4] Lang F Bohmer C Palmada M Seebohm G Strutz-Seebohm N Vallon V (Patho)physiological significance of the serum- and glucocorticoid-inducible kinase isoforms. Physiol Rev (2006) 86:1151–78. 10.1152/physrev.00050.2005 17015487

[5] Maiyar AC Leong ML Firestone GL Importin-alpha mediates the regulated nuclear targeting of serum- and glucocorticoid-inducible protein kinase (Sgk) by recognition of a nuclear localization signal in the kinase central domain. Mol Biol Cell (2003) 14:1221–39. 10.1091/mbc.e02-03-0170 PMC151592 12631736

[6] Talarico C Dattilo V D’Antona L Menniti M Bianco C Ortuso F SGK1, the New Player in the Game of Resistance: Chemo-Radio Molecular Target and Strategy for Inhibition. Cell Physiol Biochem (2016) 39:1863–76. 10.1159/000447885 27771704

[7] Lang F Artunc F Vallon V The physiological impact of the serum and glucocorticoid-inducible kinase SGK1. Curr Opin Nephrol Hypertens (2009) 18:439–48. 10.1097/MNH.0b013e32832f125e PMC2883450 19584721

[8] Valinsky WC Touyz RM Shrier A Aldosterone, SGK1, and ion channels in the kidney. Clin Sci (2018) 132:173–83. 10.1042/CS20171525 PMC5817097 29352074

[9] Pirat C Dacquet C Leclerc V Hennuyer N Beucher-Gaudin M Zanirato G Anti-diabetic activity of fused PPARgamma-SIRT1 ligands with limited body-weight gain by mimicking calorie restriction and decreasing SGK1 expression. Eur J Med Chem (2017) 137:310–26. 10.1016/j.ejmech.2017.06.006 28609708

[10] Wu C Chen Z Xiao S Thalhamer T Madi A Han T SGK1 Governs the Reciprocal Development of Th17 and Regulatory T Cells. Cell Rep (2018) 22:653–65. 10.1016/j.celrep.2017.12.068 PMC5826610 29346764

[11] Lai Y Luo XY Guo HJ Wang SY Xiong J Yang SX PO-322 has potent immunosuppressive activity in vitro and in vivo by selectively inhibiting SGK1 activity. Br J Pharmacol (2019) 13:14926. 10.1111/bph.14926 PMC7060359 31724152

[12] Di Cristofano A SGK1: The Dark Side of PI3K Signaling. Curr Top Dev Biol (2017) 123:49–71. 10.1016/bs.ctdb.2016.11.006 28236975 PMC5658788

[13] Kobayashi T Deak M Morrice N Cohen P Characterization of the structure and regulation of two novel isoforms of serum- and glucocorticoid-induced protein kinase. Biochem J (1999) 1:189–97. 10.1042/bj3440189 PMC1220630 10548550

[14] Sahoo S Brickley DR Kocherginsky M Conzen SD Coordinate expression of the PI3-kinase downstream effectors serum and glucocorticoid-induced kinase (SGK-1) and Akt-1 in human breast cancer. Eur J Cancer (2005) 41:2754–9. 10.1016/j.ejca.2005.07.018 16246546

[15] Yao Y Jiang Q Jiang L Wu J Zhang Q Wang J Lnc-SGK1 induced by Helicobacter pylori infection and highsalt diet promote Th2 and Th17 differentiation in human gastric cancer by SGK1/Jun B signaling. Oncotarget (2016) 7:20549–60. 10.18632/oncotarget.7823 PMC4991474 26942879

[16] Tang Z Shen Q Xie H Zhou Z Shi G Zhang C Serum and glucocorticoid-regulated kinase 1 (SGK1) is a predictor of poor prognosis in non-small cell lung cancer, and its dynamic pattern following treatment with SGK1 inhibitor and gamma-ray irradiation was elucidated. Oncol Rep (2018) 39:1505–15. 10.3892/or.2018.6181 29328462

[17] Xiaobo Y Qiang L Xiong Q Zheng R Jianhua Z Zhifeng L Serum and glucocorticoid kinase 1 promoted the growth and migration of non-small cell lung cancer cells. Gene (2016) 576:339–46. 10.1016/j.gene.2015.10.072 26548813

[18] Szmulewitz RZ Chung E Al-Ahmadie H Daniel S Kocherginsky M Razmaria A Serum/glucocorticoid-regulated kinase 1 expression in primary human prostate cancers. Prostate (2012) 72:157–64. 10.1002/pros.21416 PMC6000822 21563193

[19] Liu W Wang X Liu Z Wang Y Yin B Yu P SGK1 inhibition induces autophagy-dependent apoptosis via the mTOR-Foxo3a pathway. Br J Cancer (2017) 117:1139–53. 10.1038/bjc.2017.293 PMC5674106 29017179

[20] Liu W Wang X Wang Y Dai Y Xie Y Ping Y SGK1 inhibition-induced autophagy impairs prostate cancer metastasis by reversing EMT. J Exp Clin Cancer Res (2018) 37:018–0743. 10.1186/s13046-018-0743-1 PMC5879613 29609629

[21] Lang F Stournaras C Serum and glucocorticoid inducible kinase, metabolic syndrome, inflammation, and tumor growth. Hormones (2013) 12:160–71. 10.14310/horm.2002.1401 23933686

[22] Lehrer S Rheinstein PH Rosenzweig KE Glioblastoma Multiforme: Fewer Tumor Copy Number Segments of the SGK1 Gene Are Associated with Poorer Survival. Cancer Genomics Proteom (2018) 15:273–8. 10.21873/cgp.20085 PMC6070715 29976632

[23] Ronchi CL Sbiera S Leich E Tissier F Steinhauer S Deutschbein T Low SGK1 expression in human adrenocortical tumors is associated with ACTH-independent glucocorticoid secretion and poor prognosis. J Clin Endocrinol Metab (2012) 97:2012–669. 10.1210/jc.2012-2669 PMC3579951 23055545

[24] Segditsas S Sieber O Deheragoda M East P Rowan A Jeffery R Putative direct and indirect Wnt targets identified through consistent gene expression changes in APC-mutant intestinal adenomas from humans and mice. Hum Mol Genet (2008) 17:3864–75. 10.1093/hmg/ddn286 PMC2638572 18782851

[25] Wu S Wu F Jiang Z Identification of hub genes, key miRNAs and potential molecular mechanisms of colorectal cancer. Oncol Rep (2017) 38:2043–50. 10.3892/or.2017.5930 PMC5652954 28902367

[26] Ronchi CL Leich E Sbiera S Weismann D Rosenwald A Allolio B Single nucleotide polymorphism microarray analysis in cortisol-secreting adrenocortical adenomas identifies new candidate genes and pathways. Neoplasia (2012) 14:206–18. 10.1593/neo.111758 PMC3323898 22496620

[27] Ryan RJ Drier Y Whitton H Cotton MJ Kaur J Issner R Detection of Enhancer-Associated Rearrangements Reveals Mechanisms of Oncogene Dysregulation in B-cell Lymphoma. Cancer Discov (2015) 5:1058–71. 10.1158/2159-8290.CD-15-0370 PMC4592453 26229090

[28] Schuhmacher B Bein J Rausch T Benes V Tousseyn T Vornanen M JUNB, DUSP2, SGK1, SOCS1 and CREBBP are frequently mutated in T-cell/histiocyte-rich large B-cell lymphoma. Haematologica (2019) 104:330–7. 10.3324/haematol.2018.203224 PMC6355500 30213827

[29] Chen X Gu J Wu Y Liang P Shen M Xi J Clinical characteristics of colorectal cancer patients and anti-neoplasm activity of genistein. BioMed Pharmacother (2020) 124:17. 10.1016/j.biopha.2020.109835 31958764

[30] Conza D Mirra P Cali G Tortora T Insabato L Fiory F The SGK1 inhibitor SI113 induces autophagy, apoptosis, and endoplasmic reticulum stress in endometrial cancer cells. J Cell Physiol (2017) 232:3735–43. 10.1002/jcp.25850 28177128

[31] Abbruzzese C Mattarocci S Pizzuti L Mileo AM Visca P Antoniani B Determination of SGK1 mRNA in non-small cell lung cancer samples underlines high expression in squamous cell carcinomas. J Exp Clin Cancer Res (2012) 31:1756–9966. 10.1186/1756-9966-31-4 PMC3284423 22240294

[32] Simon P Schneck M Hochstetter T Koutsouki E Mittelbronn M Merseburger A Differential regulation of serum- and glucocorticoid-inducible kinase 1 (SGK1) splice variants based on alternative initiation of transcription. Cell Physiol Biochem (2007) 20:715–28. 10.1159/000110432 17982254

[33] Naruse T Yanamoto S Okuyama K Yamashita K Omori K Nakao Y Therapeutic implication of mTORC2 in oral squamous cell carcinoma. Oral Oncol (2017) 65:23–32. 10.1016/j.oraloncology.2016.12.012 28109464

[34] Melhem A Yamada SD Fleming GF Delgado B Brickley DR Wu W Administration of glucocorticoids to ovarian cancer patients is associated with expression of the anti-apoptotic genes SGK1 and MKP1/DUSP1 in ovarian tissues. Clin Cancer Res (2009) 15:3196–204. 10.1158/1078-0432.CCR-08-2131 PMC4707040 19383827

[35] O’Neill D Jones D Wade M Grey J Nakjang S Guo W Development and exploitation of a novel mutant androgen receptor modelling strategy to identify new targets for advanced prostate cancer therapy. Oncotarget (2015) 6:26029–40. 10.18632/oncotarget.4347 PMC4694883 26267320

[36] Schmid E Stagno MJ Yan J Schleicher S Yu W Honisch S Serum and Glucocorticoid Inducible Kinase 1-Sensitive Survival, Proliferation and Migration of Rhabdomyosarcoma Cells. Cell Physiol Biochem (2017) 43:1301–8. 10.1159/000481842 28992614

[37] Lessi F Beggs A de Palo M Anti M Macarone Palmieri R Francesconi S Down-regulation of serum/glucocorticoid regulated kinase 1 in colorectal tumours is largely independent of promoter hypermethylation. PLoS One (2010) 5:0013840. 10.1371/journal.pone.0013840 PMC2974649 21079778

[38] Hartmann S Schuhmacher B Rausch T Fuller L Doring C Weniger M Highly recurrent mutations of SGK1, DUSP2 and JUNB in nodular lymphocyte predominant Hodgkin lymphoma. Leukemia (2016) 30:844–53. 10.1038/leu.2015.328 26658840

[39] Fagerli UM Ullrich K Stuhmer T Holien T Kochert K Holt RU Serum/glucocorticoid-regulated kinase 1 (SGK1) is a prominent target gene of the transcriptional response to cytokines in multiple myeloma and supports the growth of myeloma cells. Oncogene (2011) 30:3198–206. 10.1038/onc.2011.79 21478911

[40] Hodgson A Amemiya Y Seth A Djordjevic B Parra-Herran C High-grade Mullerian Adenosarcoma: Genomic and Clinicopathologic Characterization of a Distinct Neoplasm With Prevalent TP53 Pathway Alterations and Aggressive Behavior. Am J Surg Pathol (2017) 41:1513–22. 10.1097/PAS.0000000000000907 28834809

[41] Kennedy BM Harris RE Cyclooxygenase and lipoxygenase gene expression in the inflammogenesis of breast cancer. Inflammopharmacology (2018) 7:018–0489. 10.1007/s10787-018-0489-6 29736687

[42] Zhang Z Xu Q Song C Mi B Zhang H Kang H Serum- and Glucocorticoid-inducible Kinase 1 is Essential for Osteoclastogenesis and Promotes Breast Cancer Bone Metastasis. Mol Cancer Ther (2020) 19:650–60. 10.1158/1535-7163.MCT-18-0783 31694887

[43] Lee LYW Woolley C Starkey T Biswas S Mirshahi T Bardella C Serum- and Glucocorticoid-induced Kinase Sgk1 Directly Promotes the Differentiation of Colorectal Cancer Cells and Restrains Metastasis. Clin Cancer Res (2019) 25:629–40. 10.1158/1078-0432.CCR-18-1033 PMC6339518 30322876

[44] Ueki S Fujishima F Kumagai T Ishida H Okamoto H Takaya K GR, Sgk1, and NDRG1 in esophageal squamous cell carcinoma: their correlation with therapeutic outcome of neoadjuvant chemotherapy. BMC Cancer (2020) 20:020–6652. 10.1186/s12885-020-6652-7 PMC7045479 32106831

[45] Isikbay M Otto K Kregel S Kach J Cai Y Vander Griend DJ Glucocorticoid receptor activity contributes to resistance to androgen-targeted therapy in prostate cancer. Horm Cancer (2014) 5:72–89. 10.1007/s12672-014-0173-2 24615402 PMC4440041

[46] Hanahan D Weinberg RA Hallmarks of cancer: the next generation. Cell (2011) 144:646–74. 10.1016/j.cell.2011.02.013 21376230

[47] Nasir O Wang K Foller M Gu S Bhandaru M Ackermann TF Relative resistance of SGK1 knockout mice against chemical carcinogenesis. IUBMB Life (2009) 61:768–76. 10.1002/iub.209 19548318

[48] Liang X Lan C Jiao G Fu W Long X An Y Therapeutic inhibition of SGK1 suppresses colorectal cancer. Exp Mol Med (2017) 49:184. 10.1038/emm.2017.184 PMC5704191 29170478

[49] Sherk AB Frigo DE Schnackenberg CG Bray JD Laping NJ Trizna W Development of a small-molecule serum- and glucocorticoid-regulated kinase-1 antagonist and its evaluation as a prostate cancer therapeutic. Cancer Res (2008) 68:7475–83. 10.1158/0008-5472.CAN-08-1047 PMC2562281 18794135

[50] Tian S Wang X Proud CG Oncogenic MNK signalling regulates the metastasis suppressor NDRG1. Oncotarget (2017) 8:46121–35. 10.18632/oncotarget.17555 PMC5542254 28545025

[51] Schmidt EM Gu S Anagnostopoulou V Alevizopoulos K Foller M Lang F Serum- and glucocorticoid-dependent kinase-1-induced cell migration is dependent on vinculin and regulated by the membrane androgen receptor. FEBS J (2012) 279:1231–42. 10.1111/j.1742-4658.2012.08515.x 22309306

[52] Matschke J Wiebeck E Hurst S Rudner J Jendrossek V Role of SGK1 for fatty acid uptake, cell survival and radioresistance of NCI-H460 lung cancer cells exposed to acute or chronic cycling severe hypoxia. Radiat Oncol (2016) 11:016–0647. 10.1186/s13014-016-0647-1 PMC4888512 27251632

[53] Mason JA Davison-Versagli CA Leliaert AK Pape DJ McCallister C Zuo J Oncogenic Ras differentially regulates metabolism and anoikis in extracellular matrix-detached cells. Cell Death Differ (2016) 23:1271–82. 10.1038/cdd.2016.15 PMC4947665 26915296

[54] Castel P Ellis H Bago R Toska E Razavi P Carmona FJ PDK1-SGK1 Signaling Sustains AKT-Independent mTORC1 Activation and Confers Resistance to PI3Kalpha Inhibition. Cancer Cell (2016) 30:229–42. 10.1016/j.ccell.2016.06.004 PMC4982440 27451907

[55] Heikamp EB Patel CH Collins S Waickman A Oh MH Sun IH The AGC kinase SGK1 regulates TH1 and TH2 differentiation downstream of the mTORC2 complex. Nat Immunol (2014) 15:457–64. 10.1038/ni.2867 PMC4267697 24705297

[56] Wang K Gu S Nasir O Foller M Ackermann TF Klingel K SGK1-dependent intestinal tumor growth in APC-deficient mice. Cell Physiol Biochem (2010) 25:271–8. 10.1159/000276561 20110688

[57] Feng Z Liu L Zhang C Zheng T Wang J Lin M Chronic restraint stress attenuates p53 function and promotes tumorigenesis. Proc Natl Acad Sci USA (2012) 109:7013–8. 10.1073/pnas.1203930109 PMC3345015 22509031

[58] Towhid ST Liu GL Ackermann TF Beier N Scholz W Fuchss T Inhibition of colonic tumor growth by the selective SGK inhibitor EMD638683. Cell Physiol Biochem (2013) 32:838–48. 10.1159/000354486 24081014

[59] Lang F Perrotti N Stournaras C Colorectal carcinoma cells–regulation of survival and growth by SGK1. Int J Biochem Cell Biol (2010) 42:1571–5. 10.1016/j.biocel.2010.05.016 20541034

[60] Wu W Zou M Brickley DR Pew T Conzen SD Glucocorticoid receptor activation signals through forkhead transcription factor 3a in breast cancer cells. Mol Endocrinol (2006) 20:2304–14. 10.1210/me.2006-0131 16690749

[61] Marzook H Deivendran S George B Reshmi G Santhoshkumar TR Kumar R Cytoplasmic translocation of MTA1 coregulator promotes de-repression of SGK1 transcription in hypoxic cancer cells. Oncogene (2017) 36:5263–73. 10.1038/onc.2017.19 28504714

[62] Toska E Castel P Chhangawala S Arruabarrena-Aristorena A Chan C Hristidis VC PI3K Inhibition Activates SGK1 via a Feedback Loop to Promote Chromatin-Based Regulation of ER-Dependent Gene Expression. Cell Rep (2019) 27:294–306. 10.1016/j.celrep.2019.02.111 30943409 PMC6503687

[63] Catalogna G Talarico C Dattilo V Gangemi V Calabria F D’Antona L The SGK1 Kinase Inhibitor SI113 Sensitizes Theranostic Effects of the 64CuCl2 in Human Glioblastoma Multiforme Cells. Cell Physiol Biochem (2017) 43:108–19. 10.1159/000480328 28848088

[64] Abbruzzese C Catalogna G Gallo E di Martino S Mileo AM Carosi M The small molecule SI113 synergizes with mitotic spindle poisons in arresting the growth of human glioblastoma multiforme. Oncotarget (2017) 8:110743–55. 10.18632/oncotarget.22500 PMC5762281 29340013

[65] Amato R D’Antona L Porciatti G Agosti V Menniti M Rinaldo C Sgk1 activates MDM2-dependent p53 degradation and affects cell proliferation, survival, and differentiation. J Mol Med (2009) 87:1221–39. 10.1007/s00109-009-0525-5 19756449

[66] Hong F Larrea MD Doughty C Kwiatkowski DJ Squillace R Slingerland JM mTOR-raptor binds and activates SGK1 to regulate p27 phosphorylation. Mol Cell (2008) 30:701–11. 10.1016/j.molcel.2008.04.027 18570873

[67] D’Antona L Amato R Talarico C Ortuso F Menniti M Dattilo V SI113, a specific inhibitor of the Sgk1 kinase activity that counteracts cancer cell proliferation. Cell Physiol Biochem (2015) 35:2006–18. 10.1159/000374008 25871776

[68] Talarico C D’Antona L Scumaci D Barone A Gigliotti F Fiumara CV Preclinical model in HCC: the SGK1 kinase inhibitor SI113 blocks tumor progression in vitro and in vivo and synergizes with radiotherapy. Oncotarget (2015) 6:37511–25. 10.18632/oncotarget.5527 PMC4741945 26462020

[69] Talarico C Dattilo V D’Antona L Barone A Amodio N Belviso S SI113, a SGK1 inhibitor, potentiates the effects of radiotherapy, modulates the response to oxidative stress and induces cytotoxic autophagy in human glioblastoma multiforme cells. Oncotarget (2016) 7:15868–84. 10.18632/oncotarget.7520 PMC4941283 26908461

[70] Salis O Okuyucu A Bedir A Gor U Kulcu C Yenen E Antimetastatic effect of fluvastatin on breast and hepatocellular carcinoma cells in relation to SGK1 and NDRG1 genes. Tumour Biol (2016) 37:3017–24. 10.1007/s13277-015-4119-2 26419593

[71] Abbruzzese C Matteoni S Persico M Ascione B Schenone S Musumeci F The small molecule SI113 hinders epithelial-to-mesenchymal transition and subverts cytoskeletal organization in human cancer cells. J Cell Physiol (2019) 234:22529–42. 10.1002/jcp.28816 31099037

[72] Liang X Lan C Zhou J Fu W Long X An Y Development of a new analog of SGK1 inhibitor and its evaluation as a therapeutic molecule of colorectal cancer. J Cancer (2017) 8:2256–62. 10.7150/jca.19566 PMC5560143 28819428

[73] Lorin S Hamai A Mehrpour M Codogno P Autophagy regulation and its role in cancer. Semin Cancer Biol (2013) 23:361–79. 10.1016/j.semcancer.2013.06.007 23811268

[74] Levy JMM Towers CG Thorburn A Targeting autophagy in cancer. Nat Rev Cancer (2017) 17:528–42. 10.1038/nrc.2017.53 PMC5975367 28751651

[75] Amaravadi RK Kimmelman AC Debnath J Targeting Autophagy in Cancer: Recent Advances and Future Directions. Cancer Discov (2019) 9:1167–81. 10.1158/2159-8290.CD-19-0292 PMC7306856 31434711

[76] Andres-Mateos E Brinkmeier H Burks TN Mejias R Files DC Steinberger M Activation of serum/glucocorticoid-induced kinase 1 (SGK1) is important to maintain skeletal muscle homeostasis and prevent atrophy. EMBO Mol Med (2013) 5:80–91. 10.1002/emmm.201201443 23161797 PMC3569655

[77] Li P Pan F Hao Y Feng W Song H Zhu D SGK1 is regulated by metabolic-related factors in 3T3-L1 adipocytes and overexpressed in the adipose tissue of subjects with obesity and diabetes. Diabetes Res Clin Pract (2013) 102:35–42. 10.1016/j.diabres.2013.08.009 24035040

[78] Li P Hao Y Pan FH Zhang M Ma JQ Zhu DL SGK1 inhibitor reverses hyperglycemia partly through decreasing glucose absorption. J Mol Endocrinol (2016) 56:301–9. 10.1530/JME-15-0285 27287220

[79] Singh PK Singh S Ganesh S Activation of serum/glucocorticoid-induced kinase 1 (SGK1) underlies increased glycogen levels, mTOR activation, and autophagy defects in Lafora disease. Mol Biol Cell (2013) 24:3776–86. 10.1091/mbc.e13-05-0261 PMC3861076 24131995

[80] Lu J Tan M Cai Q The Warburg effect in tumor progression: mitochondrial oxidative metabolism as an anti-metastasis mechanism. Cancer Lett (2015) 356:156–64. 10.1016/j.canlet.2014.04.001 PMC4195816 24732809

[81] Corbet C Bastien E Santiago de Jesus JP Dierge E Martherus R Vander Linden C TGFbeta2-induced formation of lipid droplets supports acidosis-driven EMT and the metastatic spreading of cancer cells. Nat Commun (2020) 11:019–14262. 10.1038/s41467-019-14262-3 PMC6978517 31974393

[82] Friedmann Angeli JP Krysko DV Conrad M Ferroptosis at the crossroads of cancer-acquired drug resistance and immune evasion. Nat Rev Cancer (2019) 19:405–14. 10.1038/s41568-019-0149-1 31101865

[83] Konieczkowski DJ Johannessen CM Garraway LA A Convergence-Based Framework for Cancer Drug Resistance. Cancer Cell (2018) 33:801–15. 10.1016/j.ccell.2018.03.025 PMC5957297 29763622

[84] Vasan N Baselga J Hyman DM A view on drug resistance in cancer. Nature (2019) 575:299–309. 10.1038/s41586-019-1730-1 31723286 PMC8008476

[85] Manning BD Toker A AKT/PKB Signaling: Navigating the Network. Cell (2017) 169:381–405. 10.1016/j.cell.2017.04.001 28431241 PMC5546324

[86] Pretre V Wicki A Inhibition of Akt and other AGC kinases: A target for clinical cancer therapy? Semin Cancer Biol (2018) 48:70–7. 10.1016/j.semcancer.2017.04.011 28473255

[87] Moniz LS Vanhaesebroeck B AKT-ing out: SGK kinases come to the fore. Biochem J (2013) 452:e11–3. 10.1042/BJ20130617 23725458

[88] Lu L Zhu F Li Y Kimpara S Hoang NM Pourdashti S Inhibition of the STAT3 target SGK1 sensitizes diffuse large B cell lymphoma cells to AKT inhibitors. Blood Cancer J (2019) 9(4):43. 10.1038/s41408-019-0203-y 30926771 PMC6441016

[89] Fraczek N Bronisz I Pietryka M Kepinska D Strzala P Mielnicka K An outline of main factors of drug resistance influencing cancer therapy. J Chemother (2016) 28:457–64. 10.1080/1120009X.2016.1218158 27545330

[90] Sommer EM Dry H Cross D Guichard S Davies BR Alessi DR Elevated SGK1 predicts resistance of breast cancer cells to Akt inhibitors. Biochem J (2013) 452:499–508. 10.1042/BJ20130342 23581296 PMC3671793

[91] Jo A Yun HJ Kim JY Lim SC Choi HJ Kang BS Prolyl isomerase PIN1 negatively regulates SGK1 stability to mediate tamoxifen resistance in breast cancer cells. Anticancer Res (2015) 35:785–94. 25667458

[92] Kim MJ Chae JS Kim KJ Hwang SG Yoon KW Kim EK Negative regulation of SEK1 signaling by serum- and glucocorticoid-inducible protein kinase 1. EMBO J (2007) 26:3075–85. 10.1038/sj.emboj.7601755 PMC1914103 17568772

[93] Stringer-Reasor EM Baker GM Skor MN Kocherginsky M Lengyel E Fleming GF Glucocorticoid receptor activation inhibits chemotherapy-induced cell death in high-grade serous ovarian carcinoma. Gynecol Oncol (2015) 138:656–62. 10.1016/j.ygyno.2015.06.033 PMC4556542 26115975

[94] D’Antona L Dattilo V Catalogna G Scumaci D Fiumara CV Musumeci F In Preclinical Model of Ovarian Cancer, the SGK1 Inhibitor SI113 Counteracts the Development of Paclitaxel Resistance and Restores Drug Sensitivity. Transl Oncol (2019) 12:1045–55. 10.1016/j.tranon.2019.05.008 PMC6545392 31163384

[95] Amato R Scumaci D D’Antona L Iuliano R Menniti M Di Sanzo M Sgk1 enhances RANBP1 transcript levels and decreases taxol sensitivity in RKO colon carcinoma cells. Oncogene (2013) 32:4572–8. 10.1038/onc.2012.470 23108393

[96] Zhu J Zhang R Yang D Li J Yan X Jin K Knockdown of Long Non-Coding RNA XIST Inhibited Doxorubicin Resistance in Colorectal Cancer by Upregulation of miR-124 and Downregulation of SGK1. Cell Physiol Biochem (2018) 51:113–28. 10.1159/000495168 30439718

[97] Zhang S Chang YY Gong YW Gao YJ Guo Q Wang YH Comprehensive analysis of microRNA-messenger RNA regulatory network in gemcitabine-resistant bladder cancer cells. J Cell Biochem (2019) 120:6347–60. 10.1002/jcb.27922 30304549

[98] Chen F Chen X Ren Y Weng G Keng PC Chen Y Radiation-induced glucocorticoid receptor promotes CD44+ prostate cancer stem cell growth through activation of SGK1-Wnt/β-catenin signaling. J Mol Med (2019) 97:1169–82. 10.1007/s00109-019-01807-8 31187175

[99] Bakhoum SF Cantley LC The Multifaceted Role of Chromosomal Instability in Cancer and Its Microenvironment. Cell (2018) 174:1347–60. 10.1016/j.cell.2018.08.027 PMC6136429 30193109

[100] Bian X Xiao YT Wu T Yao M Du L Ren S Microvesicles and chemokines in tumor microenvironment: mediators of intercellular communications in tumor progression. Mol Cancer (2019) 18:019–0973. 10.1186/s12943-019-0973-7 PMC6441155 30925930

[101] Zhuang X Zhang H Hu G Cancer and Microenvironment Plasticity: Double-Edged Swords in Metastasis. Trends Pharmacol Sci (2019) 40:419–29. 10.1016/j.tips.2019.04.005 PMC7518789 31078320

[102] Helms E Onate MK Sherman MH Fibroblast Heterogeneity in the Pancreatic Tumor Microenvironment. Cancer Discov (2020) 3:2159–8290. 10.1158/2159-8290 PMC8261791 32014869

[103] Binnewies M Roberts EW Kersten K Chan V Fearon DF Merad M Understanding the tumor immune microenvironment (TIME) for effective therapy. Nat Med (2018) 24:541–50. 10.1038/s41591-018-0014-x PMC5998822 29686425

[104] Hida K Maishi N Sakurai Y Hida Y Harashima H Heterogeneity of tumor endothelial cells and drug delivery. Adv Drug Delivery Rev (2016) 99:140–7. 10.1016/j.addr.2015.11.008 26626622

[105] Piperigkou Z Karamanos NK Dynamic Interplay between miRNAs and the Extracellular Matrix Influences the Tumor Microenvironment. Trends Biochem Sci (2019) 44:1076–88. 10.1016/j.tibs.2019.06.007 31288968

[106] Nagarsheth N Wicha MS Zou W Chemokines in the cancer microenvironment and their relevance in cancer immunotherapy. Nat Rev Immunol (2017) 17:559–72. 10.1038/nri.2017.49 PMC5731833 28555670

[107] Logan RM Stringer AM Bowen JM Yeoh AS Gibson RJ Sonis ST The role of pro-inflammatory cytokines in cancer treatment-induced alimentary tract mucositis: pathobiology, animal models and cytotoxic drugs. Cancer Treat Rev (2007) 33:448–60. 10.1016/j.ctrv.2007.03.001 17507164

[108] Du YN Tang XF Xu L Chen WD Gao PJ Han WQ SGK1-FoxO1 Signaling Pathway Mediates Th17/Treg Imbalance and Target Organ Inflammation in Angiotensin II-Induced Hypertension. Front Physiol (2018) 9:1581. 10.3389/fphys.2018.01581 30524295 PMC6262360

[109] Arlauckas SP Garren SB Garris CS Kohler RH Oh J Pittet MJ Arg1 expression defines immunosuppressive subsets of tumor-associated macrophages. Theranostics (2018) 8:5842–54. 10.7150/thno.26888 PMC6299430 30613266

[110] Amato R Menniti M Agosti V Boito R Costa N Bond HM IL-2 signals through Sgk1 and inhibits proliferation and apoptosis in kidney cancer cells. J Mol Med (2007) 85:707–21. 10.1007/s00109-007-0205-2 17571248

[111] Murakami Y Hosoi F Izumi H Maruyama Y Ureshino H Watari K Identification of sites subjected to serine/threonine phosphorylation by SGK1 affecting N-myc downstream-regulated gene 1 (NDRG1)/Cap43-dependent suppression of angiogenic CXC chemokine expression in human pancreatic cancer cells. Biochem Biophys Res Commun (2010) 396:376–81. 10.1016/j.bbrc.2010.04.100 20416281

[112] Zhou Y Fei M Zhang G Liang WC Lin W Wu Y Blockade of the Phagocytic Receptor MerTK on Tumor-Associated Macrophages Enhances P2X7R-Dependent STING Activation by Tumor-Derived cGAMP. Immunity (2020) 52:357–73. 10.1016/j.immuni.2020.01.014 32049051

[113] Katara GK Jaiswal MK Kulshrestha A Kolli B Gilman-Sachs A Beaman KD Tumor-associated vacuolar ATPase subunit promotes tumorigenic characteristics in macrophages. Oncogene (2014) 33:5649–54. 10.1038/onc.2013.532 24362525

[114] Tong Y Zhou L Yang L Guo P Cao Y Qin FX Concomitant type I IFN and M-CSF signaling reprograms monocyte differentiation and drives pro-tumoral arginase production. EBioMedicine (2019) 39:132–44. 10.1016/j.ebiom.2018.11.062 PMC6354658 30528455

[115] Ross SH Cantrell DA Signaling and Function of Interleukin-2 in T Lymphocytes. Annu Rev Immunol (2018) 36:411–33. 10.1146/annurev-immunol-042617-053352 PMC6472684 29677473

[116] Pearce LR Sommer EM Sakamoto K Wullschleger S Alessi DR Protor-1 is required for efficient mTORC2-mediated activation of SGK1 in the kidney. Biochem J (2011) 436:169–79. 10.1042/BJ20102103 21413931

[117] Schmidt KM Hellerbrand C Ruemmele P Michalski CW Kong B Kroemer A Inhibition of mTORC2 component RICTOR impairs tumor growth in pancreatic cancer models. Oncotarget (2017) 8:24491–505. 10.18632/oncotarget.15524 PMC5421865 28445935

[118] Hall BA Kim TY Skor MN Conzen SD Serum and glucocorticoid-regulated kinase 1 (SGK1) activation in breast cancer: requirement for mTORC1 activity associates with ER-alpha expression. Breast Cancer Res Treat (2012) 135:469–79. 10.1007/s10549-012-2161-y PMC3891577 22842983

[119] Zou JX Guo L Revenko AS Tepper CG Gemo AT Kung HJ Androgen-induced coactivator ANCCA mediates specific androgen receptor signaling in prostate cancer. Cancer Res (2009) 69:3339–46. 10.1158/0008-5472.CAN-08-3440 19318566

[120] Yemelyanov A Bhalla P Yang X Ugolkov A Iwadate K Karseladze A Differential targeting of androgen and glucocorticoid receptors induces ER stress and apoptosis in prostate cancer cells: a novel therapeutic modality. Cell Cycle (2012) 11:395–406. 10.4161/cc.11.2.18945 22223138 PMC3356826

[121] Shanmugam I Cheng G Terranova PF Thrasher JB Thomas CP Li B Serum/glucocorticoid-induced protein kinase-1 facilitates androgen receptor-dependent cell survival. Cell Death Differ (2007) 14:2085–94. 10.1038/sj.cdd.4402227 17932503

[122] Orlacchio A Ranieri M Brave M Arciuch VA Forde T De Martino D SGK1 Is a Critical Component of an AKT-Independent Pathway Essential for PI3K-Mediated Tumor Development and Maintenance. Cancer Res (2017) 77:6914–26. 10.1158/0008-5472.CAN-17-2105 PMC5732884 29055016

[123] De Marco C Laudanna C Rinaldo N Oliveira DM Ravo M Weisz A Specific gene expression signatures induced by the multiple oncogenic alterations that occur within the PTEN/PI3K/AKT pathway in lung cancer. PLoS One (2017) 12:e0178865. 10.1371/journal.pone.0178865 28662101 PMC5491004

[124] Kach J Long TM Selman P Tonsing-Carter EY Bacalao MA Lastra RR Selective Glucocorticoid Receptor Modulators (SGRMs) Delay Castrate-Resistant Prostate Cancer Growth. Mol Cancer Ther (2017) 16:1680–92. 10.1158/1535-7163.MCT-16-0923 PMC5544558 28428441

[125] Di Cecilia S Zhang F Sancho A Li S Aguiló F Sun Y RBM5-AS1 Is Critical for Self-Renewal of Colon Cancer Stem-like Cells. Cancer Res (2016) 76:5615–27. 10.1158/0008-5472.CAN-15-1824 PMC5050123 27520449

[126] Dehner M Hadjihannas M Weiske J Huber O Behrens J Wnt signaling inhibits Forkhead box O3a-induced transcription and apoptosis through up-regulation of serum- and glucocorticoid-inducible kinase 1. J Biol Chem (2008) 283:19201–10. 10.1074/jbc.M710366200 18487207

[127] Tangir J Bonafé N Gilmore-Hebert M Henegariu O Chambers SK SGK1, a potential regulator of c-fms related breast cancer aggressiveness. Clin Exp Metastasis (2004) 21:477–83. 10.1007/s10585-004-4226-8 15679045

[128] Yoo G Kim T Chung C Hwang DS Lim DS The novel YAP target gene, SGK1, upregulates TAZ activity by blocking GSK3β-mediated TAZ destabilization. Biochem Biophys Res Commun (2017) 490:650–6. 10.1016/j.bbrc.2017.06.092 28634071

[129] Ma X Zhang L Song J Nguyen E Lee RS Rodgers SJ Characterization of the Src-regulated kinome identifies SGK1 as a key mediator of Src-induced transformation. Nat Commun (2019) 10:296. 10.1038/s41467-018-08154-1 30655532 PMC6336867

[130] Godbole M Togar T Patel K Dharavath B Yadav N Janjuha S Up-regulation of the kinase gene SGK1 by progesterone activates the AP-1-NDRG1 axis in both PR-positive and -negative breast cancer cells. J Biol Chem (2018) 293:19263–76. 10.1074/jbc.RA118.002894 PMC6298595 30337371

[131] Tang L Yu W Wang Y Li H Shen Z Anlotinib inhibits synovial sarcoma by targeting GINS1: a novel downstream target oncogene in progression of synovial sarcoma. Clin Transl Oncol (2019) 21:1624–33. 10.1007/s12094-019-02090-2 30963468

[132] Yoon JW Gilbertson R Iannaccone S Iannaccone P Walterhouse D Defining a role for Sonic hedgehog pathway activation in desmoplastic medulloblastoma by identifying GLI1 target genes. Int J Cancer (2009) 124:109–19. 10.1002/ijc.23929 PMC3889649 18924150

[133] Srivastava M Leighton X Starr J Eidelman O Pollard HB Diverse effects of ANXA7 and p53 on LNCaP prostate cancer cells are associated with regulation of SGK1 transcription and phosphorylation of the SGK1 target FOXO3A. BioMed Res Int (2014) 193635:22. 10.1155/2014/193635 PMC4016907 24864229

[134] Gao D Wan L Wei W Phosphorylation of Rictor at Thr1135 impairs the Rictor/Cullin-1 complex to ubiquitinate SGK1. Protein Cell (2010) 1:881–5. 10.1007/s13238-010-0123-x PMC3374330 21204013

[135] Gao D Wan L Inuzuka H Berg AH Tseng A Zhai B Rictor forms a complex with Cullin-1 to promote SGK1 ubiquitination and destruction. Mol Cell (2010) 39:797–808. 10.1016/j.molcel.2010.08.016 20832730 PMC2939073

[136] Arora VK Schenkein E Murali R Subudhi SK Wongvipat J Balbas MD Glucocorticoid receptor confers resistance to antiandrogens by bypassing androgen receptor blockade. Cell (2013) 155:1309–22. 10.1016/j.cell.2013.11.012 PMC3932525 24315100

[137] Greenawalt EJ Edmonds MD Jain N Adams CM Mitra R Eischen CM Targeting of SGK1 by miR-576-3p Inhibits Lung Adenocarcinoma Migration and Invasion. Mol Cancer Res (2019) 17:289–98. 10.1158/1541-7786.MCR-18-0364 PMC6318035 30257988

[138] Zheng C Li X Qian B Feng N Gao S Zhao Y The lncRNA myocardial infarction associated transcript-centric competing endogenous RNA network in non-small-cell lung cancer. Cancer Manag Res (2018) 10:1155–62. 10.2147/CMAR.S163395 PMC5958945 29795987

[139] Liu T Yu T Hu H He K Knockdown of the long non-coding RNA HOTTIP inhibits colorectal cancer cell proliferation and migration and induces apoptosis by targeting SGK1. BioMed Pharmacother (2018) 98:286–96. 10.1016/j.biopha.2017.12.064 29274585

[140] Ackermann TF Boini KM Beier N Scholz W Fuchss T Lang F EMD638683, a novel SGK inhibitor with antihypertensive potency. Cell Physiol Biochem (2011) 28:137–46. 10.1159/000331722 21865856

[141] Ortuso F Amato R Artese A D’Antona L Costa G Talarico C In silico identification and biological evaluation of novel selective serum/glucocorticoid-inducible kinase 1 inhibitors based on the pyrazolo-pyrimidine scaffold. J Chem Inf Model (2014) 54:1828–32. 10.1021/ci500235f 24896223

[142] Mohammad T Siddiqui S Shamsi A Alajmi MF Hussain A Islam A Virtual Screening Approach to Identify High-Affinity Inhibitors of Serum and Glucocorticoid-Regulated Kinase 1 among Bioactive Natural Products: Combined Molecular Docking and Simulation Studies. Molecules (2020) 25:823. 10.3390/molecules25040823 PMC7070812 32070031

[143] Liu G Honisch S Schmidt S Pantelakos S Alkahtani S Toulany M Inhibition of SGK1 enhances mAR-induced apoptosis in MCF-7 breast cancer cells. Cancer Biol Ther (2015) 16:52–9. 10.4161/15384047.2014.986982 PMC4622018 25427201

[144] Berdel HO Yin H Liu JY Grochowska K Middleton C Yanasak N Targeting serum glucocorticoid-regulated kinase-1 in squamous cell carcinoma of the head and neck: a novel modality of local control. PLoS One (2014) 9:e113795. 10.1371/journal.pone.0113795 25485633 PMC4259465

[145] Wang M Xue Y Shen L Qin P Sang X Tao Z Inhibition of SGK1 confers vulnerability to redox dysregulation in cervical cancer. Redox Biol (2019) 24:20. 10.1016/j.redox.2019.101225 PMC6536746 31136958

[146] Mansley MK Wilson SM Effects of nominally selective inhibitors of the kinases PI3K, SGK1 and PKB on the insulin-dependent control of epithelial Na+ absorption. Br J Pharmacol (2010) 161:571–88. 10.1111/j.1476-5381.2010.00898.x PMC2990156 20880397

[147] Matteoni S Abbruzzese C Matarrese P De Luca G Mileo AM Miccadei S The kinase inhibitor SI113 induces autophagy and synergizes with quinacrine in hindering the growth of human glioblastoma multiforme cells. J Exp Clin Cancer Res (2019) 38:019–1212. 10.1186/s13046-019-1212-1 PMC6525441 31101126

